# 3D Printing Optimization for Environmental Sustainability: Experimenting with Materials of Protective Face Shield Frames

**DOI:** 10.3390/ma14216595

**Published:** 2021-11-02

**Authors:** Kristína Zgodavová, Kristína Lengyelová, Peter Bober, José Alberto Eguren, Amaia Moreno

**Affiliations:** 1 Faculty of Materials, Metallurgy and Recycling, Technical University of Košice, 042 00 Košice, Slovakia; 2 Faculty of Electrical Engineering and Informatics, Technical University of Košice, 042 00 Košice, Slovakia; peter.bober@tuke.sk; 3 Faculty of Engineering, Mondragon University, 20500 Arrasate, Spain; jaeguren@mondragon.edu (J.A.E.); amaia.moreno@alumni.mondragon.edu (A.M.)

**Keywords:** Design for Six Sigma, fractional factorial design, fused deposition modelling, PLA, PETG, PHA, environmental sustainability

## Abstract

The motivation for research on 3D printing of protective face shields was the urgent societal demand for healthcare in the fight against the spread of COVID19 pandemic. Research is based on a literature review that shows that objects produced by additive technologies do not always have consistent quality suitable for the given purpose of use. Besides, they have different effects on the environment and leave different footprints. The overall goal of the research was to find out the most suitable thermoplastic material for printing shield frames in terms of mechanical properties, geometric accuracy, weight, printing time, filament price, and environmental sustainability. Fused deposition modeling (FDM) technology was used for 3D printing, and three different filaments were investigated: polylactic acid (PLA), polyethylene terephthalate (PETG), and polyhydroxyalkanoate (PHA). The weighted sum method for multi-objective optimization was used. Finally, PHA material was chosen, mainly due to its environmental sustainability, as it has the most negligible impact on the environment.

## 1. Introduction

Social development is significantly driven by the impact of how new technologies are evolving and expanding. Many of them directly affect our daily lives. One of the massively deployed newer technologies is three-dimensional (3D) printing. With each new technology, new, as yet unexplored, and unknown horizons emerge [1]. Unlike subtractive manufacturing technologies, 3D printing can create any complex shape from the micro to macro dimension, allowing products to be designed for different areas of life. As with subtractive manufacturing technologies, 3D-printed products flow is from the invention of new ideas through the search for an application to the transformation into production, implementation, and disposal. Quality control of the additive manufacturing process is, according to [2] one of the most important technological requirements, especially in the medical [3] and aerospace [4] industries.

At present, there is already an extensive range of available 3D technologies and materials, and several of them may be genuinely viable in the future, with significant variability in material quality, dimensional accuracy, surface quality, as well as post-processing requirements [5].

The development and use of 3D technologies, especially those printing from plastic materials, generate an enormous amount of waste, which must then be taken care of. Sangkham findings [6] show that the number of face masks and shields used has increased with the number of confirmed SARS-CoV-2 cases. Therefore, promoting cleaner production requires a key emphasis on the safety and disposal of the final product. Power consumption seems to be the most significant environmental impact of 3D printing, but disposal of used products also requires additional energy and material. The effectiveness of replacing mass production with 3D printing depends on the process to be replaced, the 3D-printing technology, the required production volumes, and the type of material used [7]. Therefore, organizations proactively and forcibly are increasingly interested in the environment in general and in recovery, recycling, and waste disposal processes. This growing interest is also reflected in the creation of new legislation and market trends [8], which can also be generalized to waste from additive production.

In the following chapters, we present the research aimed at finding the optimal 3D printer settings and selecting a suitable material for printing protective shield frames from three different thermoplastic materials using fused deposition modeling (FDM) technology, which at the time of the research are used to print protective shields for healthcare in the fight against coronavirus COVID19. The research uses the Design for Six Sigma (DFSS) methodology for additive manufacturing [9] with modified Define–Measure–Experiment–Analyze–Verify phases according to [10].

## 2. Context and Objectives of the Study

During the corona crisis, face shields were printed for medical needs and various personal use. According to [11,12], some glasses help protect the wearer’s eyes from splashes, sprays, and droplets, while a face shield can help reduce exposure to both the eyes and other facial areas [13]. Safety visors with clear lenses and marked with the number 3 to indicate protections against droplets or splashes of liquids may be considered suitable for use against COVID19. Fused deposition modeling technology was used with an affordable Prusa I3 MK3S+ printer [14]. FDM technology works on the additive principle by stacking material on top of each other in several layers. In this process, the plastic fiber filament is pushed through a heated extrusion nozzle, moving in three-dimensional space with X, Y, and Z axes. The driver is specific to each printer. After heating the nozzle to a defined temperature, the plastic melts and is extruded onto the substrate at a precisely defined temperature. The molten material is used to form layers which then form the final product or parts thereof.

Face shields were printed at the beginning of the pandemic from polylactic acid (PLA) then from polyethylene terephthalate (PETG) filaments and were used only once and then discarded because the virus’s behavior was utterly unknown. Although at the beginning of the COVID19 pandemic, the speed of delivery of face shields was decisive, later it was necessary to think about the effectiveness of 3D printing and the impact of waste on the environment. Therefore, we also included polyhydroxyalkanoate (PHA), which is biodegradable, in the research.

This study presents partial results obtained from measurements carried out in Technical University in Košice. Measurements were taken in Neksten, s.r.o. laboratory. Communication with Mondragon University Laboratory took place through remote laboratory experimentation [15] and was continuously organized via the web.

Objectives of our study are to:

- Analyze and identify the optimum settings for the printer and each of the three materials: Prusament PLA Galaxy Silver, Prusament PETG Orange for PPE, and PHA BioWOOD Rosa3D;
- Compare materials and select the best one from the effectiveness and environmental sustainability point of view, using several optimization criteria.

In 3D printing, similarly to injection molding, according to [16], the following indicators are monitored: performance, production efficiency, and end-user reliability. The challenge is to achieve a high level for all three within the required timeframe and budget. In our case, which concerns the pandemic situation, the monitored indicators were:

- End-user reliability: flexibility, accuracy, weight.
- Production efficiency: printing time, the material (filament) price.
- Environmental sustainability: CO_2_ emissions for energy consumed.

Thirty-two samples from each material were printed for the first experiment, and then 16 samples for the second experiment to achieve the stated research objectives.

## 3. Materials

Three types of plastic filaments, PLA Prusament Galaxy Silver, PETG Prusament Orange for PPE [17], and PHA BioWOOD Rosa 3D [18], were used in the examination. The production of plastics has taken on enormous proportions since its discovery more than 100 years ago, and it is mainly associated with the issue of disposability, respectively biodegradability. Bioplastics are biodegradable or bio-based plastics produced from renewable sources. In terms of disposability of plastics, the European Union Commission Regulation No. 1357/2014 [19] applies. Single-use plastics, or disposable plastics, are used only once before they are thrown away or recycled. Our society produces hundreds of millions of tons of plastic every year, most of which cannot be recycled [20].

All three tested materials are food-contact and hygiene safe and are suitable for use in protective shields according to EN 166, 2002 [21].

### 3.1. Polylactic Acid

PLA is a thermoplastic polyester that has become a popular material for 3D printing because it is economically produced from renewable sources [22]. It is biodegradable under specific conditions because it is of natural origin (e.g., corn, sugar cane, or potatoes). Adding materials to the PLA such as wood particles, gypsum, bronze, steel, mixed filaments can be obtained. A specific feature of PLA is that when the printing temperature is lower than 225 °C, the resulting surface is glossy, but when it is higher than 225–230 °C, the surface is matte [23].

For our study, Prusament PLA Galaxy Silver filament was used. It is a proprietary product of the Prusa Research group and has a guaranteed filament diameter deviation of ±0.02 mm and high color fastness [24]. PLA resin identification code (RIC) is 7 “others” —complicated to recycle [25].

### 3.2. Polyethylene Terephthalate

PETG is a modified version of polyethylene terephthalate (PET), where “G” means “modified glycol” that is added to the material composition during polymerization. The result is a fiber that is clearer and less brittle. PETG filament combines the properties of acrylonitrile butadiene styrene (ABS) and PLA material. This thermoplastic filament is stronger and more resistant to higher temperatures. The adhesion between the layers is usually very good, the risk of twisting or significant shrinkage is small, and its recyclability is also an advantage [23]. Many manufacturers and users agree that it is a more complex printing material that requires experimentation with the 3D-printing process setup parameters. PET has a much higher processing temperature than PETG, which becomes sticky when heated, while PET remains solid. Therefore, PETG materials must be effectively sorted before recycling [26]. The disposability of PETG is more problematic than that of PLA.

For the study, Prusament PETG Orange PPE was used. The manufacturer intended this filament for the printing of protective equipment and began to produce it in response to the acute shortage of protective equipment. The guaranteed diameter deviation is ±0.03 mm, and its winding is not as precise as standard Prusaments (Prusa Research, 2020) [17]. PETG RIC’s number is 1 “recyclable and renewable” [27].

### 3.3. Polyhydroxyalkanoate

PHA is also a thermoplastic material and can be processed on conventional processing equipment. Depending on its composition, PHA is malleable and relatively elastic. Individual PHAs differ in their properties according to their chemical composition. It has the same basic properties as PLA, but they are usually better biodegradable and easy to print. They are especially suitable for large or detailed models that will not be exposed to high temperatures above 60 °C.

PHA BioWOOD Rosa 3D is made from 100% natural and renewable biopolymer [18]. It decomposes in a natural environment without oxygen and water, compost for up to 5 weeks (based on our experiment). PHA BioWOOD Rosa 3D is food certified and smells like natural wood. It was included in the testing mainly due to its rapid biodegradability

## 4. Methods

The philosophical perspective of Six Sigma views all work as processes that can be defined, measured, analyzed, improved, and controlled. Design for Six Sigma (DFSS) is used when no process exists or when an existing process is considered inadequate and requires replacement. Standard DFSS is a preventive and proactive methodology, and it is one of the most widely used in designing and determining the stability of new products or processes [9]. A specific procedure for planning and implementing design of experiments (DOE) according to [27] and life cycle assessment (LCA) methodology according to [28] was added to the DFSS road map in our research. 3D printing DOE is a procedure that tries to model a complex problem simply and, by conducting experiments or tests, gain knowledge about how the causes of the problem affect results. LCA is the factual analysis of a product’s entire life cycle in terms of sustainability [29].

### 4.1. Design for Six Sigma

New projects usually have three objectives: customer performance, efficiency for the producer, and end-user reliability. These three objectives have several indicators, but the indicator of environmental sustainability rarely belongs among them.

**Figure 1:**
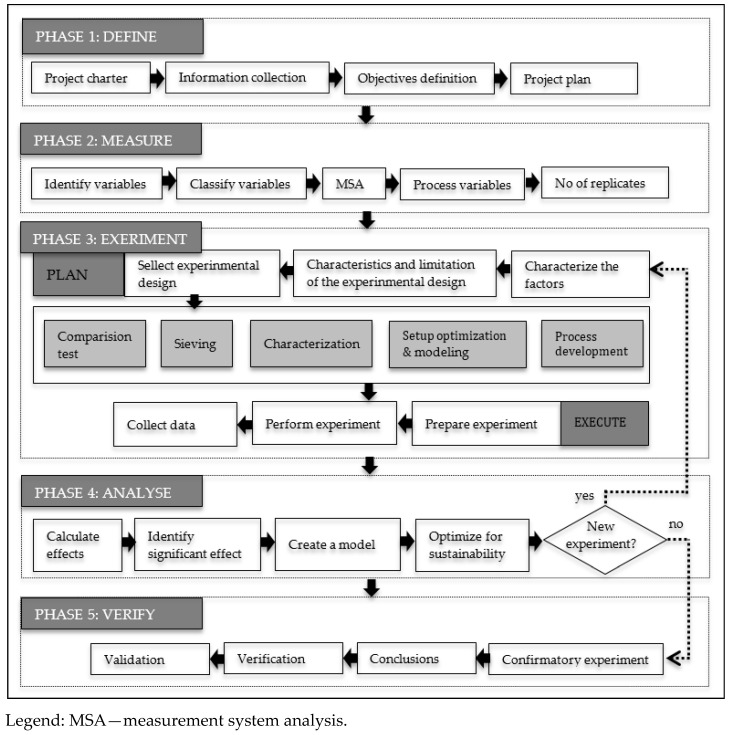
3D printing DMEAV road map.

The standard DFSS process comprises five steps: Define–Measure–Analyze–Design–Verify (DMADV). Our methodology modifies this procedure for our 3D-printing process on Define–Measure–Experiment–Analyze–Verify (DMEAV), with the Experiment step having two sub-steps, namely Experiment Planning and Experiment Execution as described in [27] and regarding environmental sustainability (Figure 1).

**Phase 1—Define:** The work team, the process, and related information, and the objective of the experimentation are defined. The team must be composed of members familiar with the process for analysis and who can identify the factors that can influence the response. Usually, the collection of information consists of identifying the parameters of the regular operation of the process.

**Phase 2—Measure:** The project team translates the customer needs and wants into measurable design requirements during the measurement phase. The team should identify and classify process variables, define the measurement system and the number of replicates. Moreover, process factors that affect the observed output should be identified and classified to obtain the maximum information that allows for minimal experimentation effort.

Factors are classified as controllable and non-controllable factors. The controllable factors include those the experimenter can consciously modify regarding the level of functioning in each experiment. For the factors identified as non-controllable, a strategy must be defined to reduce their influence and attempt to keep them constant.

**Phase 3—Experiment:** The appropriate experimental design should be selected depending on the characteristics of the process. According to [27], there are different options presented in Figure 1 based on the objective pursued to:

- Compare different situations; a comparison test would be carried out;
- Analyze the process, and when there is a limitation in the number of experiments to be executed, a sieving design is used to discard the less influential factors;
- Determine the influence of a certain number of factors with sufficient availability of resources, a characterization based on factorial designs is carried out;
- Optimize and model the process with significant factors at more than two levels, and response surface methodology is used.

It is necessary to consider the characteristics and limitations of the process and define:

- The total number of experiments that can be executed considering the constraints of the process (experimental effort);
- The number of factors, controllable and non-controllable;
- The experimental range and the levels of experimentation factors.

According to the particular objective of the study, it is necessary to prepare, implement, and collect data from the experiment and analyze them.

**Phase 4—Analysis:** The use of a regression model to predict the response for different combinations of process parameters at their best levels has proven successful in several studies [27,30,31]. The first task is to determine the regression coefficients to develop a model based on significant effects (either main or interactions). Regression coefficients are obtained for factors at two levels by dividing the effect estimates by two. The reason is that changing two units, i.e., a low-level setting (−1) to a high-level setting (+1) in the process parameter (or factor), causes a change in the function of the reaction. The effect of each factor on the response is defined as the variation of the response caused by a change in the level of the factor. By applying the methodology, it is possible to determine the process model within the experimental zone used. With the calculations completed, the coefficients of the first-order polynomial model are defined, in the form shown in Equation (1):

(1) $$\hat{y}=\beta 0+\beta 1A+\beta 2B+\beta 3C+\beta 4\mathrm{AB}+\beta 5\mathrm{AC}+\beta 6\mathrm{BC}+\ldots +\varepsilon ,$$

where β0 is the average response in a factorial experiment, β1, β2, …, βn are regression coefficients, and ε is the random error component, which is approximately normal and independently distributed with mean zero and constant variance σ^2^. The values A, B, etc., are the values that each factor or interaction takes (+1,−1). The regression coefficient β4 corresponds to the interaction between the process parameters A and B.

The measurement system must be designed and analyzed to perform correct and accurate measurements [32]. Only then can optimization be performed according to pre-agreed objectives. This phase includes a detailed high-level design for the selected alternative. Once this step is complete, the final product can be prototyped to identify where errors may occur and make necessary modifications.

**Phase 5—Verify:** In the final phase, the team validates that the design is acceptable, effective, and environmentally sustainable in the real world. Some data may lead to changes that need to be addressed so that the initial process may lead to new applications of DMADV [33]. One of the essential tools of the DFSS is the Design of Experiments.

### 4.2. Design of Experiments

The DOE state-of-the-art offers numerous established algorithms, which can be applied to various technology development tasks. However, in practice, serious obstacles often must be overcome in order to use these algorithms.

The most important methods for applying DOE include fractional and full factorial designs, ANOVA, Response Surface Methodology, and Taguchi methods [34]. In the field of analysis of properties of products created by 3D printing, [35] used Taguchi’s L27 orthogonal array for planning experiments presented by Vyavahare & Kumar in [30] choosing the full factorial design with consideration of geometric parameters for a low number of experiments, and Hoshamand et al., [31] used the response surface methodology. When the number of factors is high, the number of experiments to be performed can be high. It is possible to select a specific fraction of the complete experimental plan: one half, one quarter to reduce the number of experiments. These designs are called fractional factorial designs. Fractional factorial designs are based on the assumption that high-order interactions are negligible [36,37]. According to Box et al. [38], this design has become a widely accepted investigation method.

In a 3D-printing process, the parts produced must meet a series of mechanical and dimensional requirements. To make this possible, according to [39], a DOE has been used to optimize the parameters of the 3D printer. The methodology described in [27] is carried out to address the DOE. We also included an environmental sustainability approach in this methodology using LCA.

### 4.3. Lifecycle Assessment

Life cycle assessment (LCA) is a methodology standardized by ISO 14040:2006 Amd 1:2020) [40] and represents a key tool to measure the environmental impact of products. LCA covers all product lifecycle stages, from the extraction of resources to final disposal [41]. The LCA studies differ a lot from each other in several features and analysis conditions. Currently, the LCA and design for environment (DFE) are two methods used to assess the environmental impacts of production processes [42]. Research from [43] presents a systematic literature review of LCA application in 3D printing. Recommendations for future studies and examples of LCA are in [44].

## 5. Results and Discussion

The results described in this section are the consequence of applying the Design for Six Sigma DMEAV roadmap in our study, following the steps described in Section 4.

### 5.1. DMEAV Phases

**Figure 2:**
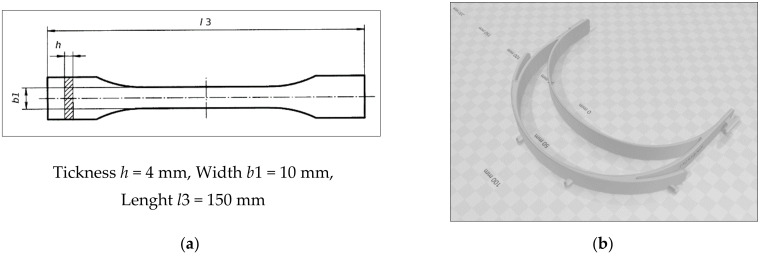
3D models: (**a**) the sample; (**b**) the face shield frame.

**Phase 1—Define:** Project charter, working team, the related information, and study objectives were defined. The team members become familiar with the defined problem, the equipment used for 3D printing, the FDM printing method, the material of filaments used, the testing and measurement equipment and process, and the final product. The shape of the sample according to ISO 527-2:2012 [45] and the face shield frame (STL file) are shown in Figure 2.

The influence of process parameters on the quality of the result must be studied to understand the performance and behavior of the FDM process [46]. The team received information that the printing process, tensile test, and dimension measurement will be performed in Neksten, s.r.o. Košice laboratories by laboratory engineer and operator, in the room with a constant temperature of 21 °C and humidity of 35%. The PLA, PETG, and PHA samples will be created with the 3D printer Original Prusa I3 MK3S+. Tensile tests will be performed according to the ISO 527-1:2019 [47] standardized procedure using the testing machine Tinius Olsen H10KS (Tinus Olsen, Horsham, PA, USA), dimensional measurements using a caliper CD-15DCX with measuring range 0–150 mm, accuracy ± 0.02 (Mitutoyo Corporation, mensional measurements using a caliper CD-15DCX, Japan), and energy consumption meter Geti GPM01 (Geti, Sobrance, Slovakia).

**Phase 2—Measure:** For this phase, the team identified process responses and factors. Initially, at the recommendation of the Mondragon University AM Lab, printer manufacturer, filament supplier, literature search, and previous team experience, 17 factors (Table S1) were identified that affect the resulting characteristics of the 3D product. Finally, five controllable factors were included in this research. The definitions of the controllable factors selected in this study are as follows:

- Layer thickness/height [mm]: The nominal layer thickness for most machines is around 0.1 mm; however, it should be noted that this is only a general principle. The reasoning is that thicker layer parts are quicker to build but are less precise.
- A number of perimeters: Defines the minimum number of outlines that form the wall of a printed product. According to [23], product strength is defined mainly by the number of perimeters.
- Extrusion width [mm]: It is a process used to create fixed cross-sectional profile objects. The filament is pushed through a die of the desired cross-section.
- Infill density [%]: Infill provides internal support for top layers, which would otherwise have to bridge over the empty space. Most products can be printed with 10-15% infill [48].
- Nozzle temperature [°C]: Temperature for melting filament. Each material has a recommended temperature.

The output responses that will be measured were also defined:

1. Flexibility:
  - Young modulus, E [GPa], according to (ISO 527-1:2019) [47] Equation (2):
    (2) $$E=\frac{\sigma }{\varepsilon }$$
    where σ is the engineering stress [MPa] and ε is a strain (elongation). The higher the young modulus, the greater the stress (greater the force with the same cross-section) to achieve the same deformation (elongation).
  - Tensile stress at break [MPa]: stress at which the specimen breaks.
  - Elongation at break [%]: Calculated as the relative increase in length Equation (3).
    (3) $$\mathrm{Elongation}\;\mathrm{at}\;\mathrm{break}=\frac{\Delta L}{L}\times 100\%$$
    where ΔL is the final length at break, and L is the initial length. The combination of high ultimate tensile strength and high elongation leads to the high toughness of materials [49].
2. Dimensional accuracy: Closeness of the measurements to a specified value.
  - Thickness [mm]
  - Width [mm]
  - Length [mm]
3. Weight: [kg]
4. Printing time: [s]
5. Material price: [€]
6. Environmental sustainability: [kg CO_2_-eq]

Carbon dioxide equivalent (CO_2_-eq) is the standardized measure for calculating the amount of greenhouse gases (GHG) emitted into the atmosphere due to a process or material use. This evolves into the Global Warming Potential (GWP) impact category, one of the main metrics used when assessing the potential impact of anything analyzed [50].

**Phase 3—Experiment:** Five controllable factors and two levels for each factor (Table 1) were defined in the previous phase. Regarding the setting of other factors, some are uncontrollable, others were set according to the manufacturer’s recommendations, and the remaining two factors are set according to Table 2. The fractional factorial design (2^5−1^) of resolution “V”, which is 1/2 fraction, was selected with design generator E = ABCD. This design allows for the analysis of the main effects and the interactions of the second order. Minitab 19 software (Minitab LLC, State College, PA, USA) created the experimental plan.

For the first experiment total of 96 samples (16 × 2 replicates × 3 materials), and for the second experiment total of 48 samples (16 × 1 replicate × 3 materials) were printed.

Regarding the selection of gyroid infill pattern, the final product (face shield) was taken into account, which is printed almost without the infill, and its pattern was in the first printing instructions. The face shield is thin, and its strength and flexibility depend more on the used material and the number of perimeters. Furthermore, according to [51], the Gyroid is Prusa printers’ favorite and one of the best infills, provides outstanding support in every direction, is printed relatively fast, saves material, and does not have crossing lines in one layer.

Measured output responses for the first experiment are mechanical properties (young modulus, tensile stress at break, elongation), accuracy (thickness, width, length), weight, printing time, and material price (Tables S2–S4). Responses for the second experiment are weight, printing time, filament price, and carbon dioxide equivalent (Tables S5–S7). Detail of the sample surface was imaged by a scanning electron microscope (SEM) JSM-IT700HR (JEOL, Tokyo, Japan).

**Figure 3:**
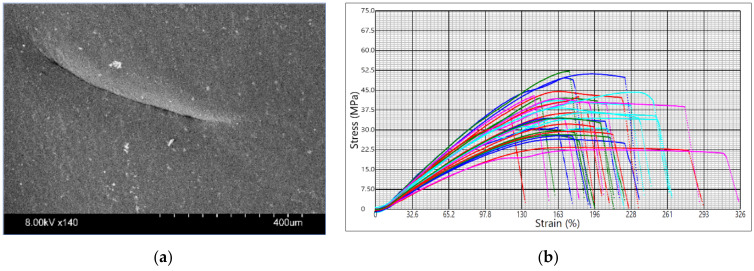
Prusament PLA Galaxy Silver: (**a**) sample surface SEM image; (**b**) tensile test graph.

**Figure 4:**
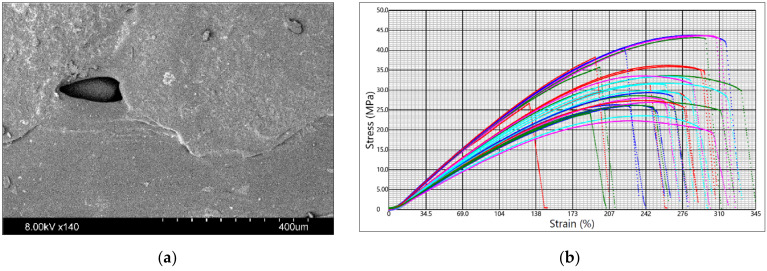
Prusament PETG Orange PPE: (**a**) sample surface SEM image; (**b**) tensile test graph.

**Figure 5:**
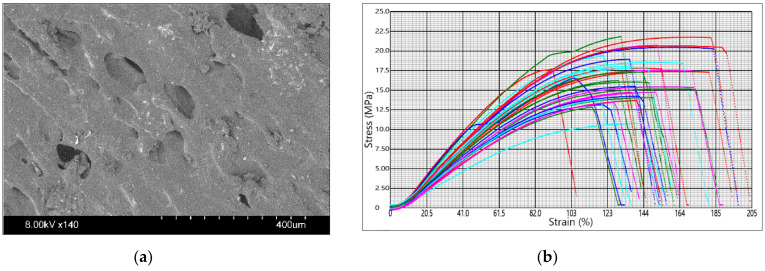
PHA BioWOOD Rosa 3D: (**a**) sample surface SEM image; (**b**) tensile test graph.

The sample images and the tensile test graphs are in Figure 3, Figure 4 and Figure 5 for PLA Galaxy Silver, PETG Orange PPE, and PHA BioWOOD Rosa, respectively. Stress–strain curves in the graphs are for different experimental settings. The figure shows the extent to which the printer settings can affect the mechanical properties of products made of the same material. The tensile test stops when the sample breaks.

Fibrous, fiber-like protrusions were observed on the surface of the specimens made of Prusament PLA Galaxy Silver, which can be explained by the material’s viscid, less viscous but sticky nature. The tensile stress at break and elongation varies from 22.5 MPa to 52.2 MPa and from 122% to 314%, respectively.

A flow pattern with a rounded end can be seen on the surface of the specimens made of PETG Orange PPE. The openings in the surface resulting from printing were irregular in shape, which may indicate rapid solidification. No foreign particles were detected. The tensile stress at break and elongation varies from 22.3 MPa to 43.8 MPa and from 132% to 332%, respectively.

In the case of plastics containing biomass particles (PHA BioWOOD Rosa), the particles can be seen even at low magnifications, their distribution is even, and they have several sizes. The plastic does not form a bond, and a gap is formed around the particles. Many small elliptical openings, which sometimes have jagged edges, are on the surface. The tensile stress at break and elongation varies from 10.8 MPa to 21.8 MPa and from 116% to 191%, respectively.

**Phase 4—Analyze:** According to the procedure in Figure 1, the obtained data are analyzed and optimized for each experiment separately. The first experiment analyzes mechanical properties and dimensional accuracy, and the second experiment analyzes environmental sustainability. Coefficients of the polynomial model were defined according to formula (1), and then analysis of variance (ANOVA), normal probability plot (NPP), main effect plot, and interaction plot were created for each response.

The *p*-value less than 0.05 in ANOVA means that the factor is statistically significant. In the NPP diagram, the main and interaction effects of the factors are plotted against the cumulative probability. Inactive main and interaction effects tend to fall roughly along a straight line, whereas active effects appear as extreme points falling off each end of the straight line [52]. These active effects are evaluated to be statistically significant. The main effect plot shows the mean response values at each design parameter or process variable level. It is used to compare the relative strength of the effects of various factors. The last interactions plot displays the mean response of two factors at all possible combinations of their settings. Horizontal lines indicate that there is no interaction between the factors.

**Figure 6:**
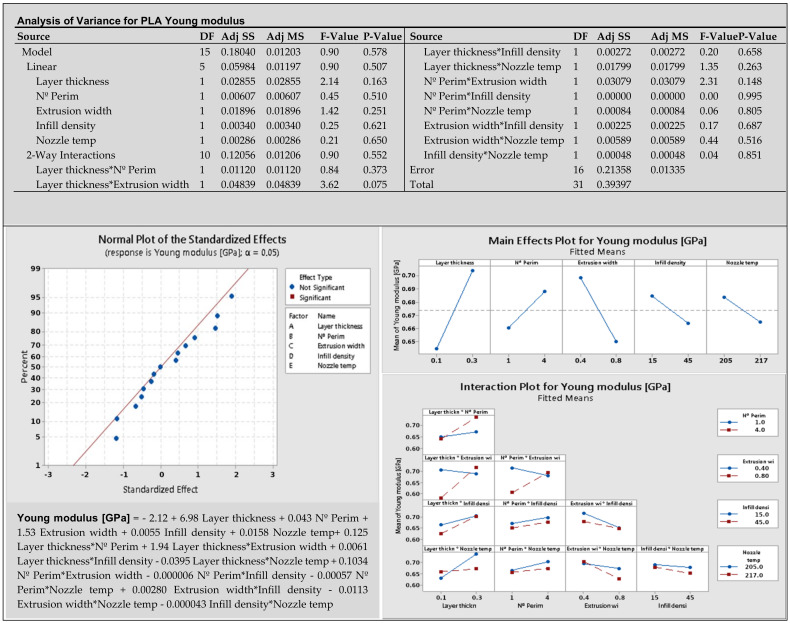
Graphical presentation of results for PLA young modulus.

Minitab 19 software was used for both analyses, which are based on calculating the effects of each factor. Graphical presentation of analysis results of selected responses (young modulus and length accuracy, PLA material) for the first experimentation with 32 samples is in Figure 6. Other figures for PETG and PHA materials are in Figures S1–S4.

**Figure 7:**
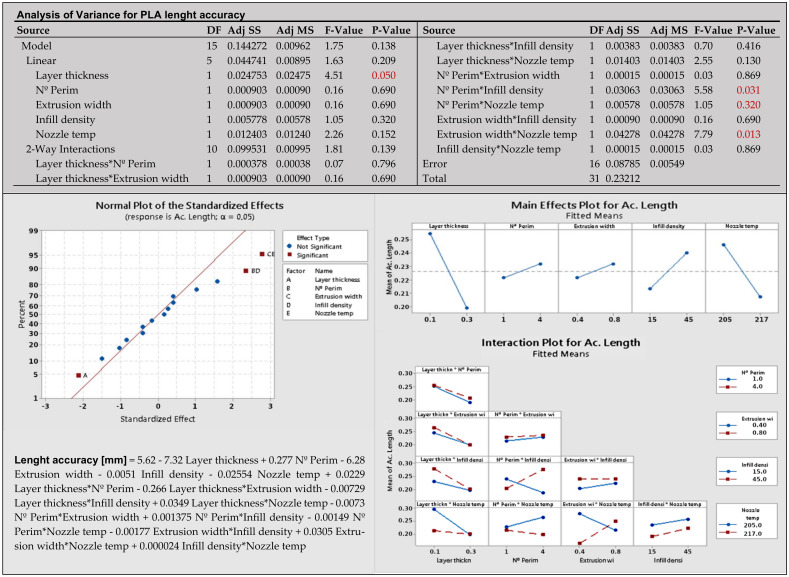
Graphical presentation of results for PLA accuracy of length.

The *p*-values of factors and interactions are listed in analysis of variance. Statistically significant factors and interactions at the significance level α = 0.05 are marked in red on the normal plot. The first normal plot for the Young modulus in Figure 6 does not contain statistically significant factors. The second normal plot for length accuracy in Figure 7 shows significant factor A—layer thickness, BD—number of perimeters*infill density, and CE—extrusion width*nozzle temperature. The main effects plot has non-horizontal lines through the X-axis for factors that are important for the response. The non-parallel lines on the interaction plot indicate the relationship between the factors. Polynomial models in Figure 6 and Figure 7 are in un-coded units and should be reduced by omitting factors and interactions with low influence.

Table 3 summarizes the result of the analysis. It contains statistically significant factors and interactions where the *p*-value is less than 0.05. Table 4 lists measured dimensional accuracy of samples printed with various setups for all three materials. The table contains absolute deviations from the nominal dimensions of the sample. The lowest overall range (marked in green) was measured for PETG and the highest (red) for PHA material.

The analysis of the first experimental data reveals that there is no clear answer for the question of which factors are most significant for all three materials. For each material, other factors are significant. There were results such as PLA young modulus that do not have any significant factors. On the other hand, there were many significant factors and interactions for PETG. These results show that each material will require an individual approach when setting the print parameters and the manufacturer’s recommendation for a given filament is not sufficient.

**Figure 8:**
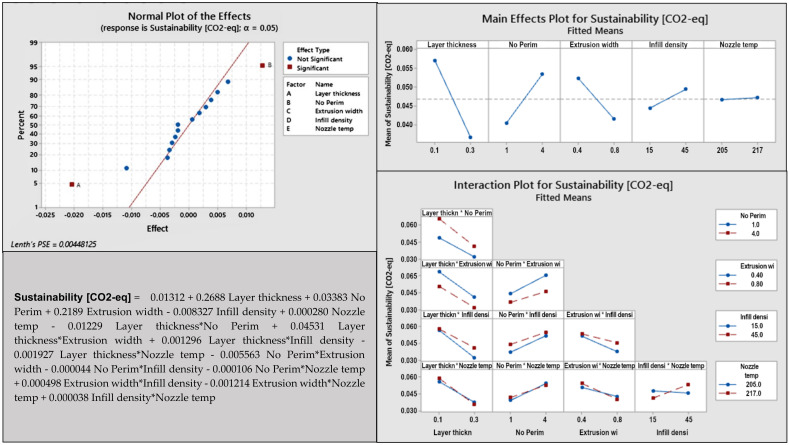
Graphical presentation of sustainability results for PLA.

After the first results were obtained, the second batch of 16 samples had been printed out. Geti GPM01 measured the consumed electrical energy for each sample. The LCA GaBi software (Sphera Solution, Leinfelden-Echterdingen, Germany) and the Ecoinvent 3.7 database (iPoint-systems, Reutlingen, Germany) were used for conversion of the energy [kWh] to CO_2_-eq [kg]. The electricity mix of Slovakia and ReCiPe method as a model for environmental impacts assessment [53] has been used. The values of the CO_2_-eq correspond only to the 3D-printing process. Results of the second analysis for PLA material are presented in Figure 8. Other figures for PETG and PHA materials are in Figures S5 and S6.

The normal plot in Figure 7 shows that the most important factors are A—layer thickness and B—number of perimeters. The main effect plot for sustainability has three non-horizontal lines through the X-axis, which are essential for the response, and include A—layer thickness, B—number of perimeters, C—extrusion width. On the interaction plot graphs, the lines are not parallel. The interaction plot indicates the relationship between DE—infill density*nozzle temperature. Table 5 summarizes the result of the analysis for all three materials.

The factor A—layer thickness is significant for all three materials. This is obvious because a thinner layer means longer printing times and higher power consumption. Factor B—number of perimeters is significant only for PLA, but factors C—extrusion width and D—infill density are significant for two materials (PETG and PHA). From the main effect plot it can be seen that the layer thickness has the highest slope being the factor with the greatest effect for all materials. The infill density and extrusion width are the second most influential for PETG and PHA. Infill density makes the printing more environmentally sustainable when it is smaller. Interestingly, in this case, the nozzle temperature does not significantly affect sustainability, although a higher temperature would appear to cause higher energy consumption.

Once the analysis has been carried out and the significance of each factor is known, the optimal level of variables was found, according to various optimization objectives in Section 5.2.

### 5.2. Optimization

Minitab 19 software uses the term individual desirability (d) for single-objective optimization and composite desirability (D) for multi-objective optimization to define the optimization objective. The desirability has a range of zero to one. Composite desirability is a weighted average of individual desirabilities.

The optimization objective for the first experiment is the composite desirability for all responses (young modulus; tensile stress at break; elongation; weight; width, thickness and length accuracy; printing time; and power consumption). All responses have the same weight value. Optimal levels of factors for each material are summarized in Table 6. Figure S7 shows the values of composite desirability and individual desirabilities for all responses.

**Figure 9:**
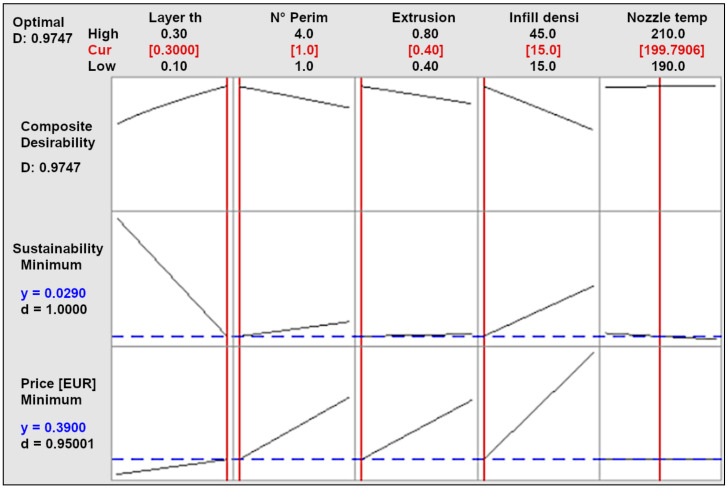
Optimization according to sustainability (CO_2_-eq) and filament price (EUR) for PHA material.

The optimization objective for the second experiment is the composite desirability consisting of sustainability and filament price. Figure 9 shows the value of composite desirability, individual desirabilities, and optimal values in red color for PHA material. Values for the remaining materials are presented in Figure S8, and optimal levels of factors for all materials are summarized in Table 7. The effect of nozzle temperature on the desirability value is small. Therefore, it is possible to set this factor to another value that will improve other properties, such as young modulus or length accuracy.

**Figure 10:**
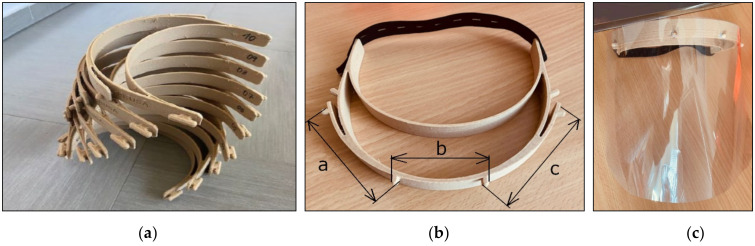
Face shield frames for PLA BioWOOD Rosa 3D filament. (**a**) the batch of printed frames; (**b**) the distance between fasting pins; (**c**) faceshield frame with counterpart.

**Phase 5—Verify:** The team validated that the final 3D printer setup for printing face shield frames is effective and environmentally sustainable. For verification, we printed a batch of 30 frames (Figure 10a) from PHA material according to Prusa Research design in Figure 2b. The printer used the optimal setup in Table 7, and the material selection is explained in Section 5.3. Measurement System Analysis (MSA) calculated by Minitab 19 shows that the measurement system’s Total Variance (TV) is less than 10%. Measurement of the thirty printed face shield frames (Figure 10a) showed that the distance between fasting pins a+b+c (Figure 10b) was in the range (−0.3, +0.3) mm, which allowed the connection with the counterpart without problem (Figure 10c).

Process capability index is one of the ways to assess the 3D-printing process performance. From the process capability index, it is possible to determine the number of non-conforming products, the production of which unnecessarily burdens the environment. This phase is not finished because measurements are not available yet.

### 5.3. Material Comparison

**Figure 11:**
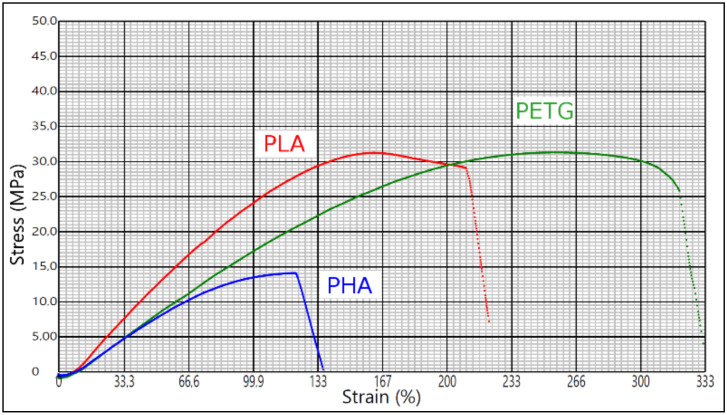
Comparison of tensile test of samples printed according to settings in Table 7.

Figure 11 shows the comparison of tensile test of samples printed according to optimal settings in Table 7.

The tensile stress at break for PLA Prusament, PETG Prusament, and PHA Bio Wood Rosa is 31.2 MPa, 31.3 MPa, and 14.1 MPa, respectively. The elongation for PLA is 210%, PETG is 320%, and PHA is 123%. In terms of flexibility, PLA is the most rigid material. The most flexible is PETG. PHA has similar flexibility but brakes first. The force required to break PETG samples is twice as high. However, this does not mean that the PHA is not applicable, but it would be necessary to consider the maximum force usually applied in face shields.

If it is necessary to make the face shield more deformable to adapt to different users, the one with the highest elongation could be selected. If the weight is important, then the PHA is the lightest. The printing time is quite similar for all three materials. However, the price makes a difference. As mentioned above, the cost of production and sustainability often do not go hand in hand, and it can be seen that the average cost of the PHA, which happens to be the most sustainable one, is double that of PLA or PETG.

## 6. Conclusions

Through this research, it was possible to complete the overall goal of the study to identify the suitability of the thermoplastic materials for the 3D printing of face shield frames using fused deposition modeling technology.

The result of the research is the design of the optimal setting of a 3D printer for three different filaments PLA Prusament, PETG Prusament, and PHA BioWOOD Rosa, from which the most suitable material in terms of environmental sustainability was then selected. The study used the DFSS methodology with modified Define—Measure—Experiment—Analyze—Verify phases so that indicators of environmental sustainability, which rarely belong to this methodology, can also be included in decision-making. Two experiments were performed, printing first 32 and then 16 samples. We selected five factors for setting up the printer (layer thickness, number of perimeters, extrusion width, infill density, and nozzle temperature) and measured outputs (mechanical properties, geometric accuracy, weight, printing time, filament price, and environmental sustainability). The analysis showed that:

- The significance of factors is different for each material and each response. Response Young modulus has no significant factor for PLA Prusament Galaxy Silver at level 0.05. There are many significant factors and interactions for PETG Prusament Orange. The most significant factors are B—number of perimeters and C—extrusion width, and interactions is BC. PHA BioWOOD Rosa 3D has the most significant interaction CD—extrusion width*infill density, and the factor is D. The situation is similar for response length accuracy. PLA Prusament has significant factor A—layer thickness and interactions BD and CE. PETG Prusament has significant interaction BE and factor B, and PHA BioWOOD has no significant factors.
- Each material requires a different optimal setting of the 3D printer for the selected objective function, and the manufacturer’s recommendation for a given filament is not sufficient.
- The most suitable material is PHA BioWOOD Rosa 3D mainly because of its sustainability, as it has the lowest impact on the environment with a mean of 0.0416 kg CO_2_-eq.
- This material is also suitable in terms of dimensional accuracy, which was verified by the experimental assembly of thirty frames with visors.
- The nozzle temperature does not significantly affect PHA BioWOOD Rosa 3D sustainability, but it affects other responses. Therefore, the temperature value in Table 7 can be replaced by a lower one to improve length accuracy.
- The prediction model also allows finding the response value for the values of factors for which no experiment was performed.

Many experiments were performed in the study, and many different parameters were measured and recorded. These data can be used to design other new products made from monitored materials on the Prusa I3 MK3S + 3D printer. The proposed DMEAV roadmap can be applied to new product development for a wide range of 3D technologies.

## Figures and Tables

**Table 1:** Selected controllable factors and levels.

Minitab Code	Factor		Experimental Level	
			(+)	(−)
A	Layer thickness [mm]	-	0.1	0.3
B	Number of perimeters	-	1	4
C	Extrusion width [mm]	-	0.4	0.8
D	Infill density [%]	-	15	45
E	Nozzle temperature [°C]	PLA	205	217
-	-	PETG	240	260
-	-	PHA	190	210

**Table 2:** Printer settings for parameters that did not change in the experiment.

Filament	PLA Prusament		PETG Prusament		PHA BioWOOD Rosa 3D	
Printer setting	recommended	Set	recommended	Set	recommended	Set
Heat bed temperature [°C]	50–60	60	70–90	90	40–50	45
Infill pattern	gyroid	Gyroid	gyroid	gyroid	gyroid	Gyroid

**Table 3:** The significant factors and interactions for young modulus and length accuracy.

Response	Materials		
	PLA Prusament	PETG Prusament	PHA BioWood
Young modulus	no significant factor	B, C, BC, D, A, AB, CD, AE, DE, AD	CD, D
Length accuracy	A, BD, CE	BE, B	no significant factor

A—layer thickness, B—number of perimeters, C—extrusion width, D—infill density, E—nozzle temperature, XY—interaction between factors X and Y.

**Table 4:** Deviations from nominal dimensions of the sample.

Measured Dimensions	Materials								
	PLA Prusament			PETG Prusament			PHA BioWood		
Dimensional deviation [mm]	**upper**	**lower**	**range**	**upper**	**lower**	**range**	**upper**	**lower**	**range**
With-nominal value 4 [mm]	+0.392	−0.002	**0.394**	+0.157	−0.092	**0.249**	+0.290	−0.031	0.321
Thickness—10 [mm]	+0.144	−0.119	**0.263**	+0.219	−0.253	0.472	+0.863	−0.495	**1.358**
Length—150 [mm]	+0.005	−0.400	0.405	+0.560	+0.210	**0.350**	+0.530	+0.110	**0.420**

**Table 5:** The most important factors and interactions for environmental sustainability.

Response	Materials		
	PLA	PETG	PHA
Environmental sustainability	A, B	A, C, D	A, C, D, AC

A—layer thickness, B—number of perimeters, C—extrusion width, D—infill density, E—nozzle temperature, XY—interaction between factors X and Y.

**Table 6:** Optimal values of 3D printer setup for PLA, PETG, and PEH according to all responses.

Code	Variable	Material		
		PLA	PETG	PHA
A	Layer thickness [mm]	0.3	0.3	0.3
B	Number of perimeters	4	4	1
C	Extrusion width [mm]	0.4	0.51	0.4
D	Infill density [%]	15	15	31.1
E	Nozzle temperature [°C]	207.8	248	210

**Table 7:** Optimal values of 3D printer setup according to sustainability and filament price.

Code	Variable	Material		
		PLA	PETG	PHA
A	Layer thickness [mm]	0.3	0.3	0.3
B	Number of perimeters	1	1	1
C	Extrusion width [mm]	0.4	0.4	0.4
D	Infill density [%]	15	15	15
E	Nozzle temperature [°C]	205	245.7	199.8

## Data Availability

Data sharing is not applicable for this article.

## Supplementary Materials

The following are available online at https://www.mdpi.com/article/10.3390/ma14216595/s1, Supplementary Table S1. All identified factors that affect responses. Table S2. Measured data for PLA for the first experiments. Table S3. Measured data for PETG for the first experiments. Table S4. Measured data for PHA for the first experiments. Figure S1. PETG Prusament Young modulus for the first experiments. Figure S2. PETG Prusament Length accuracy for the first experiments. Figure S3. PHA Bio Wood Young modulus for the first experiments. Figure S4. PHA Bio Wood Length accuracy for the first experiments. Table S5. Measured data for PLA for the second experiments. Table S6. Measured data for PETG for the second experiments. Table S7. Measured data for PHA for the second experiments. Figure S5. PETG sustainability for the second experiments. Figure S6. PHA sustainability for the second experiments. Figure S7. Composite desirability and individual desirabilities for all responses for (a) PLA, (b) PETG, (c) PHA. Figure S8. Optimization according to sustainability (CO_2_-eq) and filament price (EUR) for (a) PLA, (b) PETG material.
