# Supplementary material for: 3D Printing Optimization for Environmental Sustainability: Experimenting with Materials of Protective Face Shield Frames
Source: Materials (Basel). 2021 Nov 2;14(21):6595. doi: 10.3390/ma14216595 (PMC8585375; doi:10.3390/ma14216595)
Supplement: Supplementary file 1 [file materials-14-06595-s001.zip › materials-1383547-supplementary.pdf]

Table S1. All identified factors that affect responses.

N.	Factor	Factor type	Classification	Strategy	Expert Effect	Factor range
1	Extruder temperature	Continuous	Controllable	Control	Positive	PETG: 240-260<br>PLA: 205-220<br>PHA:190-210
2	Nozzle movement speed	Continuous	Controllable	Control	Negative	40-80 mm/s
3	Thickness of layer	Continuous	Controllable	Control	Positive	0,1 – 0,3
4	Extrusion width	Continuous	Controllable	Control	Positive	0,4 – 0,8
5	Test tube position	Discrete	Controllable	Control		Horizontal - Vertical
6	Internal infill angle	Discrete	Controllable	Control		0º - 90º
7	Nozzle type	Discrete	Controllable	Use the nozzle, recommended by the manufacturer	Null influence	0.6
8	Material	Discrete	Controllable	Use three materials	Null influence	PETG, PLA, PHA
9	Overlap – last layer perimeter	Continuous	Controllable	Use the same value	Null influence	0,15
10	First layer (speed, …)	Continuous	Controllable	Use the same value	Null influence	Established by the manufacturing Prusa Slicer program
11	Position on the table (quadrant)	Discrete	Controllable	Use the same position	Null influence	
12	Cooling	Continuous	Noise	Fans running to cool the environment inside the printer		
13	Ambient temperature	Discrete	Noise	Randomize		
14	Ambient humidity	Discrete	Noise	Randomize		
15	Humidity, raw material	Discrete	Noise	Randomize		
16	Infill pattern	Discrete	Controllable	Use the same		Gyroid, honeycomb, grid, etc.
17	Heated bed temperature	Continuous	Controllable	Control	Null influence	PETG: 70-90<br>PLA: 50-60<br>PHA:40-50

Table S2. Measured data for PLA for the first experiments.

								Flexibility			Accuracy [mm]					
Name	Std Order	Run Order	Layer thickness	Nº Perim	Extrusion width	Infill density	Nozzle temp	Young modulus [GPa]	Tensile stress at break [Mpa]	Elongation [%]	Ac. Width	Ac. Thickness	Ac. Length	Weight [g]	Printing Time [min]	Price [EUR]
01A	31	1	0.1	4	0.8	45	205	0.784	35.103	121.793	0.124	-0.227	0.25	8.56	46	0.23
02A	21	2	0.1	1	0.8	15	205	0.558	28.112	183.063	0.073	-0.085	0.22	6.30	31	0.17
03A	22	3	0.3	1	0.8	15	217	0.641	30.919	163.979	0.105	-0.160	0.37	6.52	14	0.18
04A	19	4	0.1	4	0.4	15	205	0.693	40.656	206.878	-0.119	-0.031	0.06	6.58	60	0.17
05A	18	5	0.3	1	0.4	15	205	0.559	30.628	209.276	0.062	-0.099	0.28	5.98	25	0.16
06A	30	6	0.3	1	0.8	45	205	0.674	36.469	190.782	0.109	-0.188	0.30	6.62	18	0.21
07A	29	7	0.1	1	0.8	45	217	0.733	42.050	198.351	0.028	-0.062	0.16	7.51	41	0.20
08A	23	8	0.1	4	0.8	15	217	0.855	49.577	178.717	0.063	-0.101	0.12	7.87	40	0.21
09A	25	9	0.1	1	0.4	45	205	0.611	34.289	169.327	0.032	-0.017	0.21	5.69	51	0.15
10A	28	10	0.3	4	0.4	45	205	0.741	41.080	228.370	-0.107	-0.141	0.10	7.73	34	0.20
11A	27	11	0.1	4	0.4	45	217	0.772	44.519	222.172	-0.102	-0.058	0.17	7.71	80	0.20
12A	32	12	0.3	4	0.8	45	217	0.861	42.091	147.590	0.058	-0.346	0.25	8.61	18	0.23
13A	17	13	0.1	1	0.4	15	217	0.615	34.434	206.723	0.013	-0.071	0.21	5.74	51	0.15
14A	20	14	0.3	4	0.4	15	217	0.745	40.469	276.819	-0.075	-0.176	0.09	7.80	34	0.20
15A	26	15	0.3	1	0.4	45	217	0.633	34.404	252.656	0.062	-0.160	0.26	7.28	34	0.19
16A	24	16	0.3	4	0.8	15	205	0.834	42.599	181.759	0.085	-0.381	0.28	7.96	16	0.22
17A	7	17	0.1	4	0.8	15	217	0.832	52.190	173.789	0.073	-0.122	0.10	7.90	40	0.21
18A	8	18	0.3	4	0.8	15	205	0.857	51.171	223.267	0.088	-0.392	0.25	7.99	16	0.22
19A	3	19	0.1	4	0.4	15	205	0.705	41.746	192.696	-0.056	-0.048	0.19	6.69	60	0.17
20A	12	20	0.3	4	0.4	45	205	0.666	36.434	251.358	-0.083	-0.212	0.18	7.23	34	0.20
21A	10	21	0.3	1	0.4	45	217	0.554	23.318	280.098	0.090	-0.991	0.26	7.11	34	0.19
22A	9	22	0.1	1	0.4	45	205	0.546	28.104	210.070	0.042	-0.052	0.25	4.39	76	0.19
23A	1	23	0.1	1	0.4	15	217	0.502	26.480	223.021	0.038	-0.012	0.23	5.49	51	0.15
24A	15	24	0.1	4	0.8	45	205	0.790	42.911	142.068	0.131	-0.077	0.19	8.68	46	0.23
25A	16	25	0.3	4	0.8	45	217	0.762	44.262	249.319	0.144	-0.285	0.39	8.60	18	0.23
26A	4	26	0.3	4	0.4	15	217	0.579	32.206	195.911	-0.047	-0.100	0.19	7.95	27	0.18
27A	5	27	0.1	1	0.8	15	205	0.553	29.590	178.467	0.100	-0.060	0.27	6.31	31	0.17
28A	6	28	0.3	1	0.8	15	217	0.559	27.747	177.961	0.129	-0.200	0.34	6.52	14	0.18
29A	2	29	0.3	1	0.4	15	205	0.477	22.482	313.996	0.073	-0.203	0.32	5.94	25	0.16
30A	11	30	0.1	4	0.4	45	217	0.671	38.259	237.094	0.043	0.002	0.12	7.78	80	0.20
31A	14	31	0.3	1	0.8	45	205	0.575	29.414	212.401	0.131	-0.127	0.40	6.56	18	0.21
32A	13	32	0.1	1	0.8	45	217	0.633	34.464	201.264	0.102	-0.061	0.24	7.44	41	0.20

Table S3. Measured data for PETG for the first experiments.

								Flexibility			Accuracy [mm]					
Name	Std Order	Run Order	Layer thickness	Nº Perim	Extrusion width	Infill density	Nozzle temp	Young modulus [GPa]	Tensile stress at break [Mpa]	Elongation [%]	Ac Width	Ac. Thickness	Ac. Length	Weight [g]	Printing Time [min]	Price [EUR]
01A	23	1	0.1	4	0.8	15	260	0.481	26.663	132.447	0.219	-0.085	0.41	7.83	40	0.26
02A	29	2	0.1	1	0.8	45	260	0.402	33.627	331.774	0.160	0.048	0.53	7.43	41	0.25
03A	21	3	0.1	1	0.8	15	240	0.358	26.311	227.029	0.119	0.051	0.40	6.15	31	0.21
04A	30	4	0.3	1	0.8	45	240	0.349	28.035	261.166	0.171	-0.050	0.26	7.53	20	0.25
05A	25	5	0.1	1	0.4	45	240	0.363	29.928	284.513	0.027	0.061	0.27	7.09	76	0.23
06A	20	6	0.3	4	0.4	15	260	0.365	29.542	277.979	-0.253	0.014	0.26	6.58	27	0.22
07A	26	7	0.3	1	0.4	45	260	0.345	26.960	312.208	0.086	-0.022	0.40	7.32	34	0.24
08A	32	8	0.3	4	0.8	45	260	0.484	43.763	317.944	8.996	-0.009	0.21	8.60	21	0.29
09A	24	9	0.3	4	0.8	15	240	0.476	43.660	308.376	0.150	-0.077	0.44	7.87	19	0.27
10A	18	10	0.3	1	0.4	15	240	0.310	23.549	292.596	0.111	-0.025	0.45	5.86	25	0.19
11A	19	11	0.1	4	0.4	15	240	0.376	31.692	245.892	0.078	0.028	0.41	6.50	60	0.21
12A	31	12	0.1	4	0.8	45	240	0.492	35.756	198.764	0.160	-0.067	0.43	8.40	46	0.28
13A	17	13	0.1	1	0.4	15	260	0.322	26.182	247.263	0.045	0.070	0.42	5.79	51	0.19
14A	22	14	0.3	1	0.8	15	260	0.328	26.922	269.340	0.146	0.032	0.53	6.46	17	0.22
15A	28	15	0.3	4	0.4	45	240	0.403	33.422	284.165	0.024	-0.007	0.35	7.63	35	0.25
16A	27	16	0.1	4	0.4	45	260	0.422	36.177	293.857	-0.002	0.025	0.33	7.66	81	0.25
17A	8	17	0.3	4	0.8	15	240	0.493	43.148	298.371	0.171	-0.157	0.36	7.90	19	0.27
18A	6	18	0.3	1	0.8	15	260	0.352	26.141	252.746	0.150	-0.030	0.53	6.41	17	0.22
19A	7	19	0.1	4	0.8	15	260	0.471	43.668	312.476	0.174	0.007	0.40	7.83	40	0.26
20A	4	20	0.3	4	0.4	15	260	0.366	29.492	274.867	-0.059	-0.026	0.35	6.54	27	0.22
21A	11	21	0.1	4	0.4	45	260	0.419	35.870	297.473	-0.244	0.092	0.24	7.73	81	0.25
22A	9	22	0.1	1	0.4	45	240	0.363	28.576	267.812	0.039	-0.005	0.47	6.79	77	0.23
23A	14	23	0.3	1	0.8	45	240	0.364	29.356	267.214	0.121	-0.009	0.52	7.47	20	0.25
24A	2	24	0.3	1	0.4	15	240	0.308	22.276	305.226	0.106	-0.085	0.50	5.83	25	0.19
25A	3	25	0.1	4	0.4	15	240	0.371	31.516	260.976	0.027	0.057	0.40	6.60	60	0.21
26A	10	26	0.3	1	0.4	45	260	0.350	27.372	277.890	0.065	0.004	0.40	7.15	34	0.24
27A	1	27	0.1	1	0.4	15	260	0.335	26.160	250.804	0.067	0.004	0.44	5.52	51	0.19
28A	16	28	0.3	4	0.8	45	260	0.497	40.642	223.292	-0.140	-0.037	0.43	8.58	21	0.29
29A	13	29	0.1	1	0.8	45	260	0.407	33.552	286.648	0.177	0.007	0.49	7.37	41	0.25
30A	12	30	0.3	4	0.4	45	240	0.406	31.732	318.533	0.028	-0.122	0.39	7.01	35	0.25
31A	15	31	0.1	4	0.8	45	240	0.493	38.264	195.235	0.187	-0.072	0.56	8.50	46	0.28
32A	5	32	0.1	1	0.8	15	240	0.350	24.158	190.374	0.145	-0.050	0.46	6.16	31	0.21

TableS4. Measured data for PHA for the first experiments.

								Flexibility			Accuracy [mm]					
Name	Std Order	Run Order	Layer thickness	Nº Perim	Extrusion width	Infill density	Nozzle temp	Young modulus [GPa]	Tensile stress at break [Mpa]	Elongation [%]	Ac. Width	Ac. Thickness	Ac. Length	Weight [g]	Printing Time [min]	Price [EUR]
01A	31	1	0.1	4	0.8	45	190	0.586	17.719	131.992	0.044	-0.049	-0.02	7.73	49	0.57
02A	19	2	0.1	4	0.4	15	190	0.423	16.030	146.881	-0.096	0.031	-0.11	5.85	61	0.43
03A	25	3	0.1	1	0.4	45	190	0.359	13.166	126.151	-0.112	-0.031	-0.15	6.56	79	0.47
04A	28	4	0.3	4	0.4	45	190	0.471	17.555	171.278	-0.388	-0.140	-0.52	7.10	35	0.51
05A	23	5	0.1	4	0.8	15	210	0.538	19.429	122.842	-0.042	-0.083	-0.11	7.26	42	0.52
06A	24	6	0.3	4	0.8	15	190	0.531	21.754	184.627	-0.202	-0.240	-0.17	7.39	18	0.54
07A	17	7	0.1	1	0.4	15	210	0.351	12.842	116.334	-0.330	-0.072	-0.29	5.40	52	0.38
08A	27	8	0.1	4	0.4	45	210	0.479	18.937	136.783	-0.183	0.021	-0.20	7.09	83	0.50
09A	20	9	0.3	4	0.4	15	210	0.404	14.748	138.738	-0.491	-0.136	-0.45	6.60	27	0.44
10A	18	10	0.3	1	0.4	15	190	0.326	10.784	144.245	-0.300	-0.207	-0.06	5.60	25	0.39
11A	21	11	0.1	1	0.8	15	190	0.402	17.326	131.437	0.863	-0.013	-0.07	5.94	32	0.42
12A	26	12	0.3	1	0.4	45	210	0.382	14.042	149.442	-0.189	-0.112	-0.22	6.75	35	0.48
13A	32	13	0.3	4	0.8	45	210	0.535	20.457	183.428	-0.380	-0.251	-0.42	8.05	21	0.58
14A	30	14	0.3	1	0.8	45	190	0.392	14.752	150.781	-0.131	-0.229	-0.02	7.11	20	0.51
15A	29	15	0.1	1	0.8	45	210	0.449	18.554	166.193	-0.010	-0.041	0.02	7.05	42	0.50
16A	22	16	0.3	1	0.8	15	210	0.354	13.680	142.440	-0.140	-0.195	-0.04	6.14	16	0.44
17A	7	17	0.1	4	0.8	15	210	0.556	21.831	131.014	0.027	-0.110	-0.03	7.30	42	0.52
18A	15	18	0.1	4	0.8	45	190	0.602	16.555	119.532	0.076	-0.098	0.11	7.81	49	0.57
19A	8	19	0.3	4	0.8	15	190	0.539	20.733	151.575	-0.313	-0.290	-0.27	7.43	18	0.54
20A	11	20	0.1	4	0.4	45	210	0.482	18.078	138.076	-0.225	-0.115	-0.26	7.15	83	0.50
21A	12	21	0.3	4	0.4	45	190	0.452	17.385	180.925	-0.393	-0.116	-0.35	6.70	35	0.51
22A	4	22	0.3	4	0.4	15	210	0.409	15.143	174.149	-0.476	-0.110	-0.39	6.60	27	0.44
23A	10	23	0.3	1	0.4	45	210	0.366	14.239	143.853	-0.155	-0.071	-0.18	6.56	35	0.48
24A	14	24	0.3	1	0.8	45	190	0.399	15.420	172.378	-0.092	-0.177	-0.06	7.06	20	0.51
25A	1	25	0.1	1	0.4	15	210	0.367	13.759	120.696	-0.172	0.021	-0.30	5.20	52	0.38
26A	3	26	0.1	4	0.4	15	190	0.445	17.798	154.827	-0.048	0.015	-0.23	5.95	61	0.43
27A	2	27	0.3	1	0.4	15	190	0.446	17.278	145.874	-0.047	-0.019	-0.48	5.55	25	0.39
28A	9	28	0.1	1	0.4	45	190	0.402	15.465	140.778	-0.082	-0.002	-0.18	5.27	79	0.47
29A	6	29	0.3	1	0.8	15	210	0.376	13.463	129.507	-0.074	-0.247	-0.08	6.10	16	0.44
30A	13	30	0.1	1	0.8	45	210	0.474	17.854	147.342	-0.092	-0.046	-0.27	6.97	42	0.50
31A	16	31	0.3	4	0.8	45	210	0.537	20.632	190.944	-0.417	-0.217	-0.53	8.02	21	0.58
32A	5	32	0.1	1	0.8	15	190	0.409	16.217	129.524	0.004	-0.020	-0.20	5.95	32	0.42

Analysis of Variance for PETG Young modulus

Source	DF	Adj SS	Adj MS	F-Value	P-Value
Model	15	0.11297	0.007531	143.20	0.000
Linear	5	0.10081	0.020162	383.37	0.000
Layer thickness	1	0.00164	0.001639	31.17	0.000
Nº Perim	1	0.06175	0.061754	1174.23	0.000
Extrusion width	1	0.02952	0.029523	561.37	0.000
Infill density	1	0.00774	0.007743	147.23	0.000
Nozzle temp	1	0.00015	0.000151	2.87	0.110
2-Way Interactions	10	0.01216	0.001216	23.12	0.000
Layer thickness*Nº Perim	1	0.00080	0.000799	15.19	0.001
Layer thickness*Extrusion width	1	0.00000	0.000003	0.06	0.816
Layer thickness*Infill density	1	0.00028	0.00028	5.23	0.036
Layer thickness*Nozzle temp	1	0.00041	0.00041	7.80	0.013
Nº Perim*Extrusion width	1	0.00933	0.00933	177.35	0.000
Nº Perim*Infill density	1	0.00014	0.00014	2.59	0.127
Nº Perim*Nozzle temp	1	0.00020	0.00020	3.80	0.069
Extrusion width*Infill density	1	0.00059	0.00059	11.16	0.004
Extrusion width*Nozzle temp	1	0.00002	0.00002	0.30	0.592
Infill density*Nozzle temp	1	0.000407	0.00041	7.74	0.013
Error	16	0.00084	0.0000		
Total	31	0.11381			

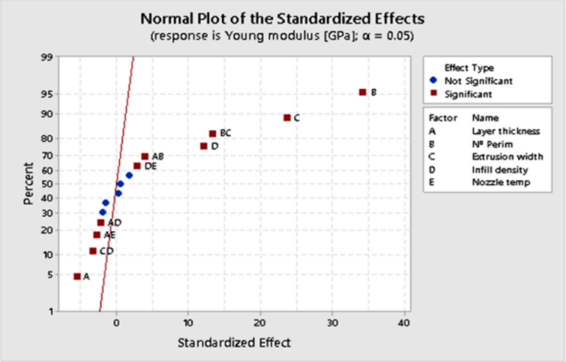

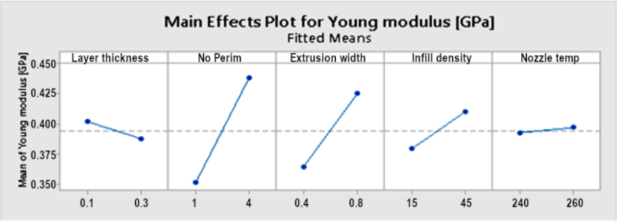

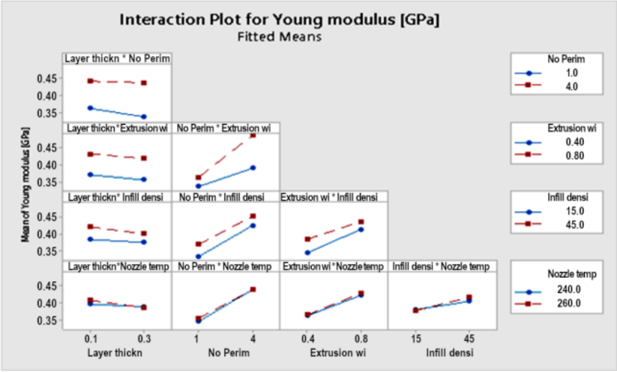

Young modulus [GPa] = 0.166 + 0.790 Layer thickness + 0.0329 Nº Perim - 0.038 Extrusion width - 0.00343 Infill density + 0.000427 Nozzle temp + 0.03331 Layer thickness*Nº Perim + 0.0151 Layer thickness*Extrusion width - 0.001954 Layer thickness*Infill density - 0.00358 Layer thickness*Nozzle temp + 0.05691 Nº Perim*Extrusion width - 0.000092 Nº Perim*Infill density - 0.000167 Nº Perim*Nozzle temp - 0.001427 Extrusion width*Infill density + 0.000350 Extrusion width*Nozzle temp + 0.000024 Infill density*Nozzle temp

Figure S1. PETG Prusament Young modulus for the first experiments.

Analysis of Variance for PETG Prusament Length accuracy

Source	DF	Adj SS	Adj MS	F-Value	P-Value
Model	15	0.14994	0.00100	1.56	0.193
Linear	5	0.07372	0.01474	2.30	0.094
Layer thickness	1	0.00236	0.00236	0.37	0.552
Nº Perim	1	0.03747	0.03747	5.85	0.028
Extrusion width	1	0.02393	0.02393	3.73	0.071
Infill density	1	0.00705	0.00705	1.10	0.310
Nozzle temp	1	0.00291	0.00291	0.45	0.510
2-Way Interactions	10	0.07622	0.00762	1.19	0.365
Layer thickness*Nº Perim	1	0.00766	0.00766	1.20	0.290
Layer thickness*Extrusion width	1	0.00861	0.00861	1.34	0.263
Layer thickness*Infill density	1	0.00591	0.00591	0.92	0.351
Layer thickness*Nozzle temp	1	0.00002	0.00002	0.00	0.957
Nº Perim*Extrusion width	1	0.00057	0.00057	0.09	0.769
Nº Perim*Infill density	1	0.00291	0.00291	0.45	0.510
Nº Perim*Nozzle temp	1	0.03955	0.03955	6.17	0.024
Extrusion width*Infill density	1	0.00236	0.00236	0.37	0.552
Extrusion width*Nozzle temp	1	0.00797	0.00797	1.24	0.281
Infill density*Nozzle temp	1	0.00066	0.00066	0.10	0.753
Error	16	0.10251	0.00641		
Total	31	0.25245			

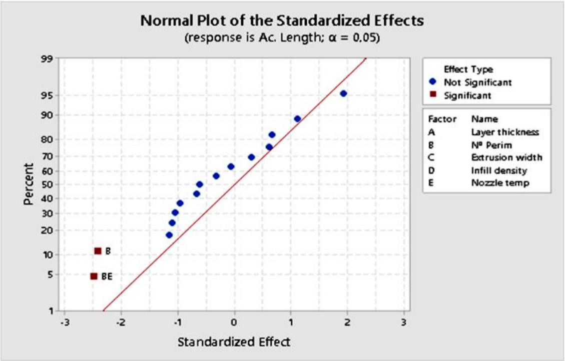

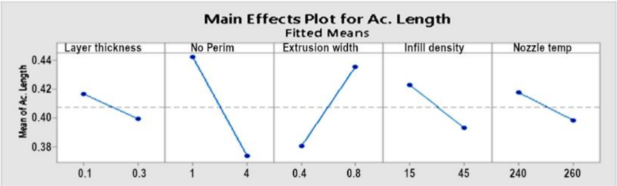

Ac. Length [mm] = 0.02 + 1.13 Layer thickness + 0.563 Nº Perim - 1.79 Extrusion width + 0.0056 Infill density + 0.00123 Nozzle temp - 0.1031 Layer thickness*Nº Perim - 0.820 Layer thickness*Extrusion width - 0.00906 Layer thickness*Infill density - 0.0008 Layer thickness*Nozzle temp + 0.0141 Nº Perim*Extrusion width + 0.000424 Nº Perim*Infill density - 0.002344 Nº Perim*Nozzle temp + 0.00286 Extrusion width*Infill density + 0.00789 Extrusion width*Nozzle temp - 0.000030 Infill density*Nozzle temp

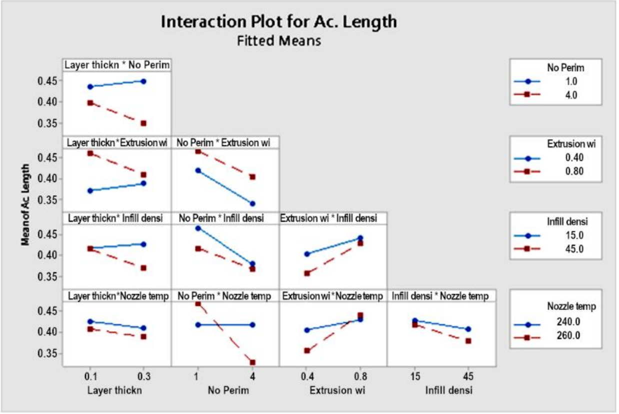

Figure S2. PETG Prusament Length accuracy for the first experiments.

Analysis of Variance for PHA Bio Wood Young modulus

Source	DF	Adj SS	Adj MS	F-Value	P-Value
Model	15	0.09806	0.00654	1.35	0.279
Linear	5	0.03691	0.00738	1.52	0.238
Layer thickness	1	0.00254	0.00254	0.52	0.479
Nº Perim	1	0.01084	0.01084	2.24	0.154
Extrusion width	1	0.00063	0.00063	0.13	0.722
Infill density	1	0.02216	0.02216	4.57	0.048
Nozzle temp	1	0.00074	0.00074	0.15	0.702
2-Way Interactions	10	0.06115	0.00612	1.26	0.327
Layer thickness*Nº Perim	1	0.00064	0.00064	0.13	0.721
Layer thickness*Extrusion width	1	0.00003	0.00003	0.01	0.935
Layer thickness*Infill density	1	0.00456	0.00456	0.94	0.347
Layer thickness*Nozzle temp	1	0.00031	0.00031	0.06	0.803
Nº Perim*Extrusion width	1	0.00059	0.00059	0.12	0.732
Nº Perim*Infill density	1	0.01575	0.01575	3.25	0.090
Nº Perim*Nozzle temp	1	0.00449	0.00449	0.93	0.350
Extrusion width*Infill density	1	0.02230	0.02230	4.60	0.048
Extrusion width*Nozzle temp	1	0.01230	0.01230	2.54	0.131
Infill density*Nozzle temp	1	0.00018	0.00018	0.04	0.851
Error	16	0.07756	0.00485		
Total	31	0.175624			

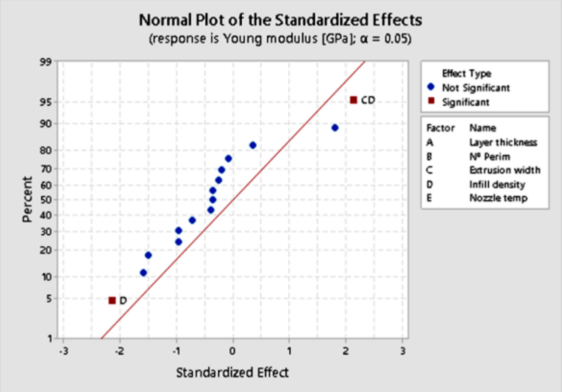

Young modulus [GPa] = -0.96 + 0.73 Layer thickness + 0.119 Nº Perim + 1.72 Extrusion width - 0.0048 Infill density + 0.00847 Nozzle temp + 0.0298 Layer thickness*Nº Perim - 0.051 Layer thickness*Extrusion width - 0.00796 Layer thickness*Infill density - 0.0031 Layer thickness*Nozzle temp - 0.0143 Nº Perim*Extrusion width + 0.000986 Nº Perim*Infill density - 0.000789 Nº Perim*Nozzle temp + 0.00880 Extrusion width*Infill density - 0.00980 Extrusion width*Nozzle temp - 0.000016 Infill density*Nozzle temp

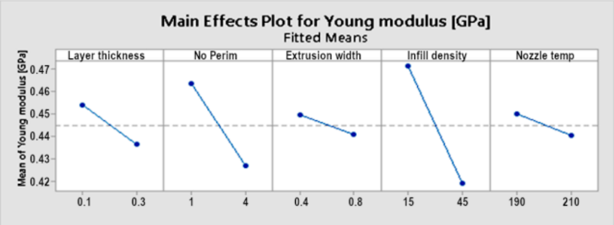

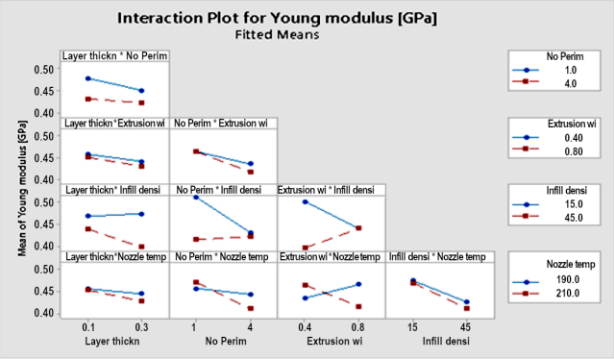

Figure S3. PHA Bio Wood Young modulus for the first experiments.

Analysis of Variance for PHA Bio Wood lenght accuracy

Source	DF	Adj SS	Adj MS	F-Value	P-Value
Model	15	0.34924	0.02328	0.75	0.710
Linear	5	0.05441	0.01088	0.35	0.875
Layer thickness	1	0.02486	0.02487	0.80	0.384
Nº Perim	1	0.01674	0.01674	0.54	0.474
Extrusion width	1	0.00008	0.00008	0.00	0.959
Infill density	1	0.00884	0.00884	0.28	0.601
Nozzle temp	1	0.00387	0.00387	0.12	0.729
2-Way Interactions	10	0.29483	0.02948	0.95	0.518
Layer thickness*Nº Perim	1	0.00387	0.00387	0.12	0.729
Layer thickness*Extrusion width	1	0.00259	0.00259	0.08	0.776
Layer thickness*Infill density	1	0.05184	0.05184	1.67	0.215
Layer thickness*Nozzle temp	1	0.06956	0.06956	2.24	0.154
Nº Perim*Extrusion width	1	0.06055	0.06055	1.95	0.182
Nº Perim*Infill density	1	0.03075	0.03075	0.99	0.335
Nº Perim*Nozzle temp	1	0.00939	0.00939	0.30	0.590
Extrusion width*Infill density	1	0.02832	0.02832	0.91	0.354
Extrusion width*Nozzle temp	1	0.00068	0.00068	0.02	0.884
Infill density*Nozzle temp	1	0.03727	0.03727	1.20	0.290
Error	16	0.49731	0.03108		
Total	1	0.05184	0.05184	1.67	0.215

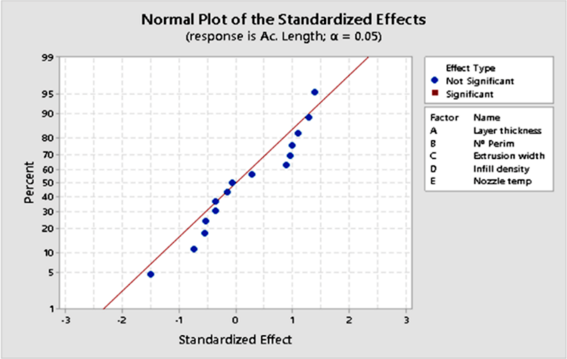

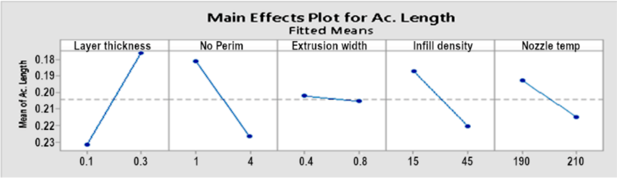

Length accuracy [mm] = 0.63 + 8.71 Layer thickness + 0.099 Nº Perim - 0.30 Extrusion width - 0.0614 Infill density + 0.0056 Nozzle temp - 0.073 Layer thickness*Nº Perim + 0.45 Layer thickness*Extrusion width + 0.0268 Layer thickness*Infill density - 0.0466 Layer thickness*Nozzle temp + 0.145 Nº Perim*Extrusion width + 0.00138 Nº Perim*Infill density - 0.00114 Nº Perim*Nozzle temp + 0.0099 Extrusion width*Infill density - 0.0023 Extrusion width*Nozzle temp + 0.000228 Infill density*Nozzle temp

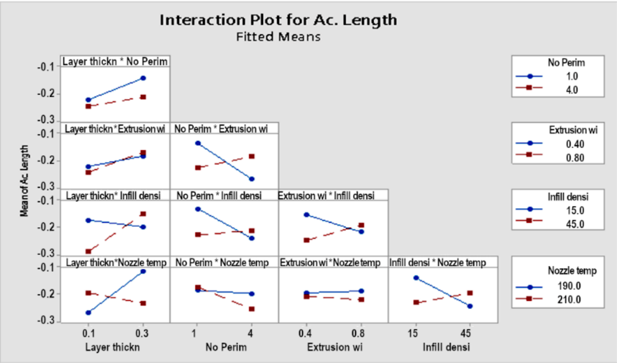

Figure S4. PHA Bio Wood Length accuracy for the first experiments.

Table S5. Measured data for PLA for the second experiments.

Name	Std Order	Run Order	Layer thickness	Nº Perim	Extrusion width	Infill density	Nozzle temp	Weight [g]	Printing Time [s]	Price [EUR]	Sustainability [CO2-eq]
01A	15	1	0.1	4	0.8	45	205	8.56	2760	0.23	0.056
02A	5	2	0.1	1	0.8	15	205	6.30	1860	0.17	0.044
03A	6	3	0.3	1	0.8	15	217	6.52	840	0.18	0.017
04A	3	4	0.1	4	0.4	15	205	6.58	3600	0.17	0.077
05A	2	5	0.3	1	0.4	15	205	5.98	1500	0.16	0.033
06A	14	6	0.3	1	0.8	45	205	6.62	1080	0.21	0.034
07A	13	7	0.1	1	0.8	45	217	7.51	2460	0.20	0.052
08A	7	8	0.1	4	0.8	15	217	7.87	2400	0.21	0.052
09A	9	9	0.1	1	0.4	45	205	5.69	3060	0.15	0.046
10A	12	10	0.3	4	0.4	45	205	7.73	2040	0.20	0.046
11A	11	11	0.1	4	0.4	45	217	7.71	4800	0.20	0.077
12A	16	12	0.3	4	0.8	45	217	8.61	1080	0.23	0.040
13A	1	13	0.1	1	0.4	15	217	5.74	3060	0.15	0.054
14A	4	14	0.3	4	0.4	15	217	7.80	2040	0.20	0.042
15A	10	15	0.3	1	0.4	45	217	7.28	2040	0.19	0.044
16A	8	16	0.3	4	0.8	15	205	7.96	960	0.22	0.037

Table S6. Measured data for PETG for the second experiments.

Name	Std Order	Run Order	Layer thickness	Nº Perim	Extrusion width	Infill density	Nozzle temp	Weight [g]	Printing Time [s]	Price [EUR]	Sustainability [CO2-eq]
01A	7	1	0.1	4	0.8	15	260	7.83	2400	0.26	0.0712
02A	13	2	0.1	1	0.8	45	260	7.43	2460	0.25	0.0686
03A	5	3	0.1	1	0.8	15	240	6.15	1860	0.21	0.0544
04A	14	4	0.3	1	0.8	45	240	7.53	1200	0.25	0.0497
05A	9	5	0.1	1	0.4	45	240	7.09	4560	0.23	0.0964
06A	4	6	0.3	4	0.4	15	260	6.58	1620	0.22	0.0518
07A	10	7	0.3	1	0.4	45	260	7.32	2040	0.24	0.0549
08A	16	8	0.3	4	0.8	45	260	8.60	1260	0.29	0.0576
09A	8	9	0.3	4	0.8	15	240	7.87	1140	0.27	0.0523
10A	2	10	0.3	1	0.4	15	240	5.86	1500	0.19	0.0481
11A	3	11	0.1	4	0.4	15	240	6.50	3600	0.21	0.0744
12A	15	12	0.1	4	0.8	45	240	8.40	2760	0.28	0.0802
13A	1	13	0.1	1	0.4	15	260	5.79	3060	0.19	0.0691
14A	6	14	0.3	1	0.8	15	260	6.46	1020	0.22	0.0472
15A	12	15	0.3	4	0.4	45	240	7.63	2100	0.25	0.0627
16A	11	16	0.1	4	0.4	45	260	7.66	4860	0.25	0.1070

Table S7. Measured data for PHA for the second experiments.

Name	Std Order	Run Order	Layer thickness	Nº Perim	Extrusion width	Infill density	Nozzle temp	Weight [g]	Printing Time [s]	Price [EUR]	Sustainability [CO2-eq]
01A	15	1	0.1	4	0.8	45	190	7.73	2940	0.57	0.0472
02A	3	2	0.1	4	0.4	15	190	5.85	3660	0.43	0.0488
03A	9	3	0.1	1	0.4	45	190	6.56	4740	0.47	0.0606
04A	12	4	0.3	4	0.4	45	190	7.10	2100	0.51	0.0377
05A	7	5	0.1	4	0.8	15	210	7.26	2520	0.52	0.0454
06A	8	6	0.3	4	0.8	15	190	7.39	1080	0.54	0.0298
07A	1	7	0.1	1	0.4	15	210	5.40	3120	0.38	0.0474
08A	11	8	0.1	4	0.4	45	210	7.09	4980	0.50	0.0636
09A	4	9	0.3	4	0.4	15	210	6.60	1620	0.44	0.0333
10A	2	10	0.3	1	0.4	15	190	5.60	1500	0.39	0.0295
11A	5	11	0.1	1	0.8	15	190	5.94	1920	0.42	0.0425
12A	10	12	0.3	1	0.4	45	210	6.75	2100	0.48	0.0369
13A	16	13	0.3	4	0.8	45	210	8.05	1260	0.58	0.0360
14A	14	14	0.3	1	0.8	45	190	7.11	1200	0.51	0.0314
15A	13	15	0.1	1	0.8	45	210	7.05	2520	0.50	0.0463
16A	6	16	0.3	1	0.8	15	210	6.14	960	0.44	0.0290

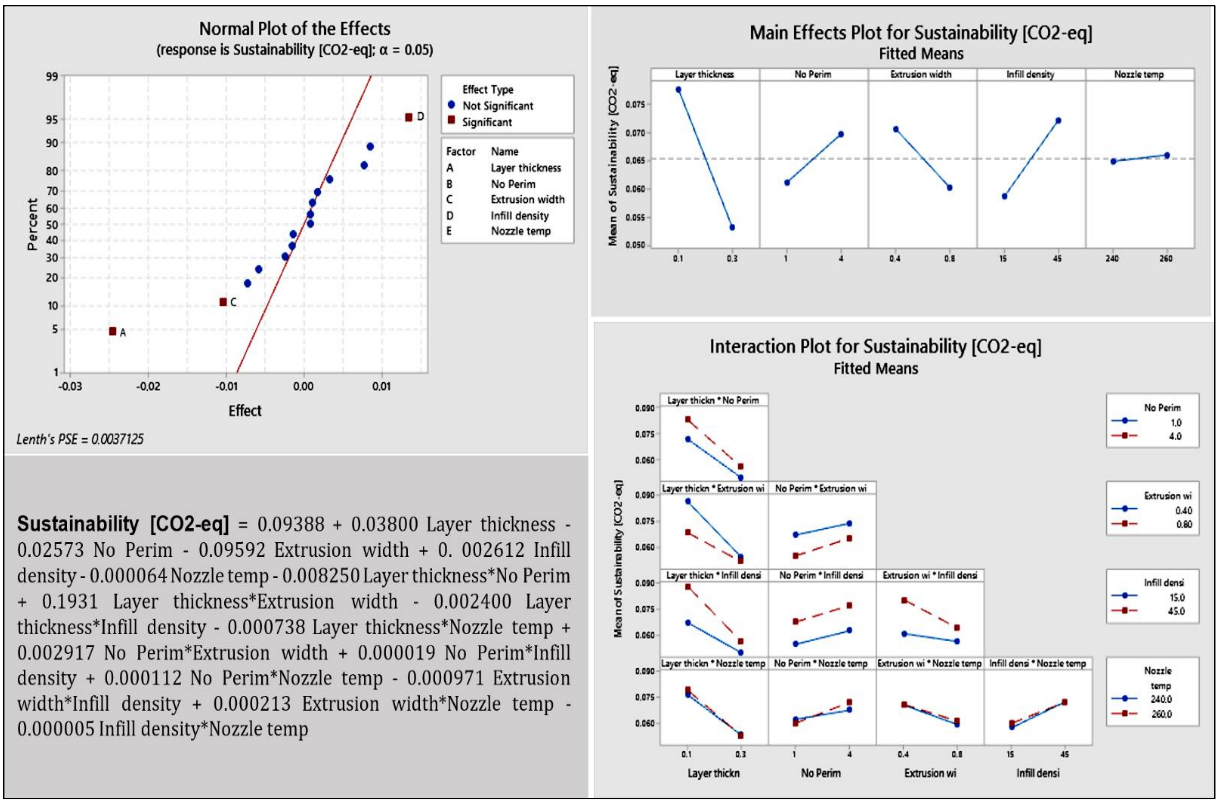

Figure S5. PETG sustainability for the second experiments.

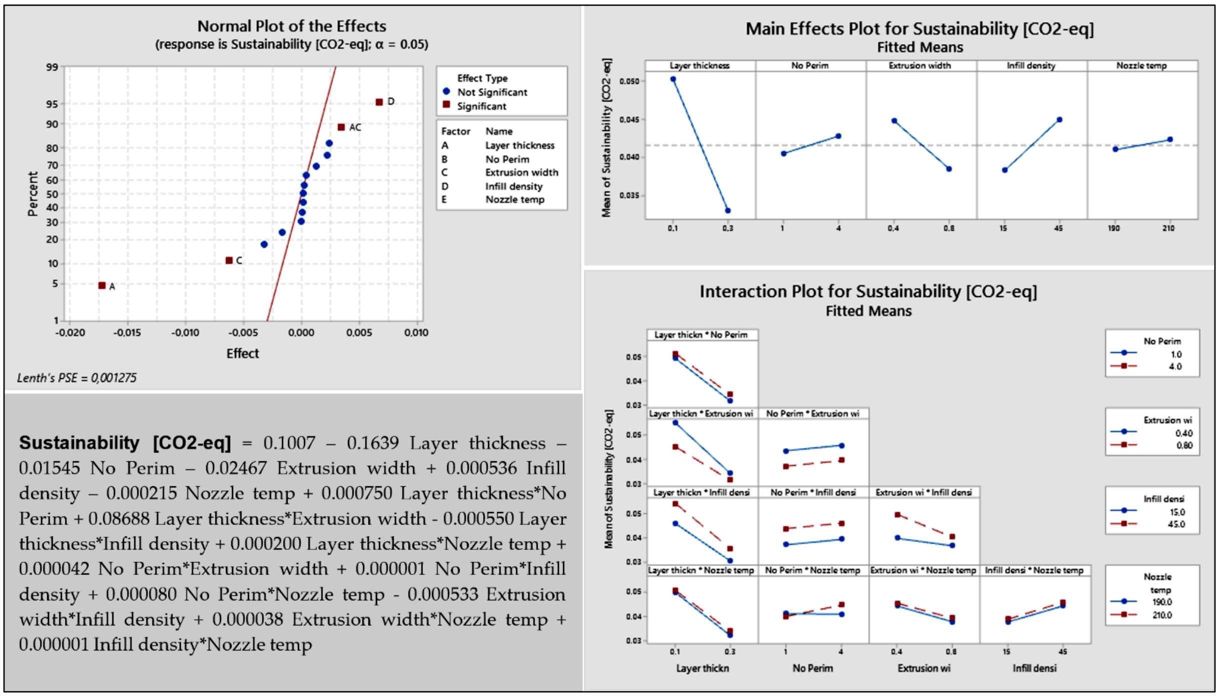

Figure S6. PHA sustainability for the second experiments.

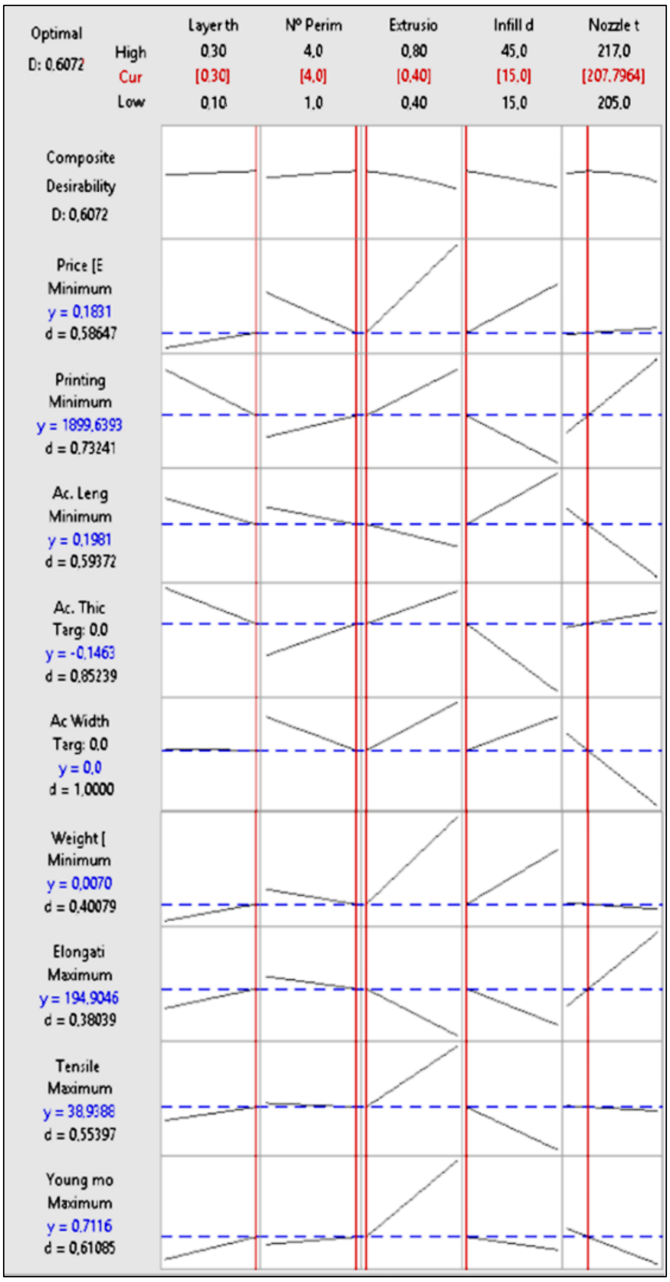
Optimal
D: 0,6072
High
Cur
Low
Layer th
0,30
[0,30]
0,10
Nº Perim
4,0
[4,0]
1,0
Extrusio
0,80
[0,40]
0,40
Infill d
45,0
[15,0]
15,0
Nozzle t
217,0
[207,7964]
205,0
Composite
Desirability
D: 0,6072
Price [E
Minimum
y = 0,1831
d = 0,58647
Printing
Minimum
y = 1899,6393
d = 0,73241
Ac. Leng
Minimum
y = 0,1981
d = 0,59372
Ac. Thic
Targ: 0,0
y = -0,1463
d = 0,85239
Ac Width
Targ: 0,0
y = 0,0
d = 1,0000
Weight [
Minimum
y = 0,0070
d = 0,40079
Elongati
Maximum
y = 194,9046
d = 0,38039
Tensile
Maximum
y = 38,9388
d = 0,55397
Young mo
Maximum
y = 0,7116
d = 0,61085

(a)

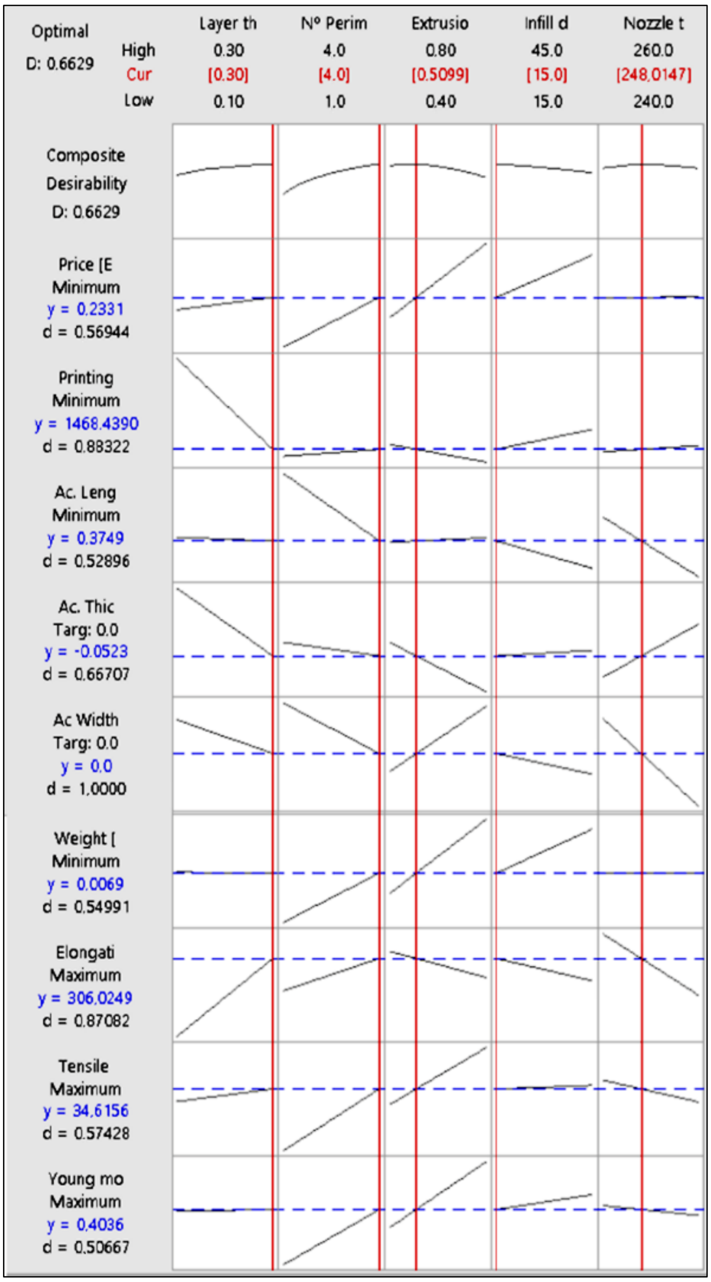

Optimal
D: 0.6629
High
Cur
Low
Layer th
0.30
[0.30]
0.10
Nº Perim
4.0
[4.0]
1.0
Extrusio
0.80
[0.5099]
0.40
Infill d
45.0
[15.0]
15.0
Nozzle t
260.0
[248.0147]
240.0
Composite Desirability
D: 0.6629
Price [E
Minimum
y = 0.2331
d = 0.56944
Printing
Minimum
y = 1468.4390
d = 0.88322
Ac. Leng
Minimum
y = 0.3749
d = 0.52896
Ac. Thic
Targ: 0.0
y = -0.0523
d = 0.66707
Ac Width
Targ: 0.0
y = 0.0
d = 1.0000
Weight [
Minimum
y = 0.0069
d = 0.54991
Elongati
Maximum
y = 306.0249
d = 0.87082
Tensile
Maximum
y = 34.6156
d = 0.57428
Young mo
Maximum
y = 0.4036
d = 0.50667

(b)

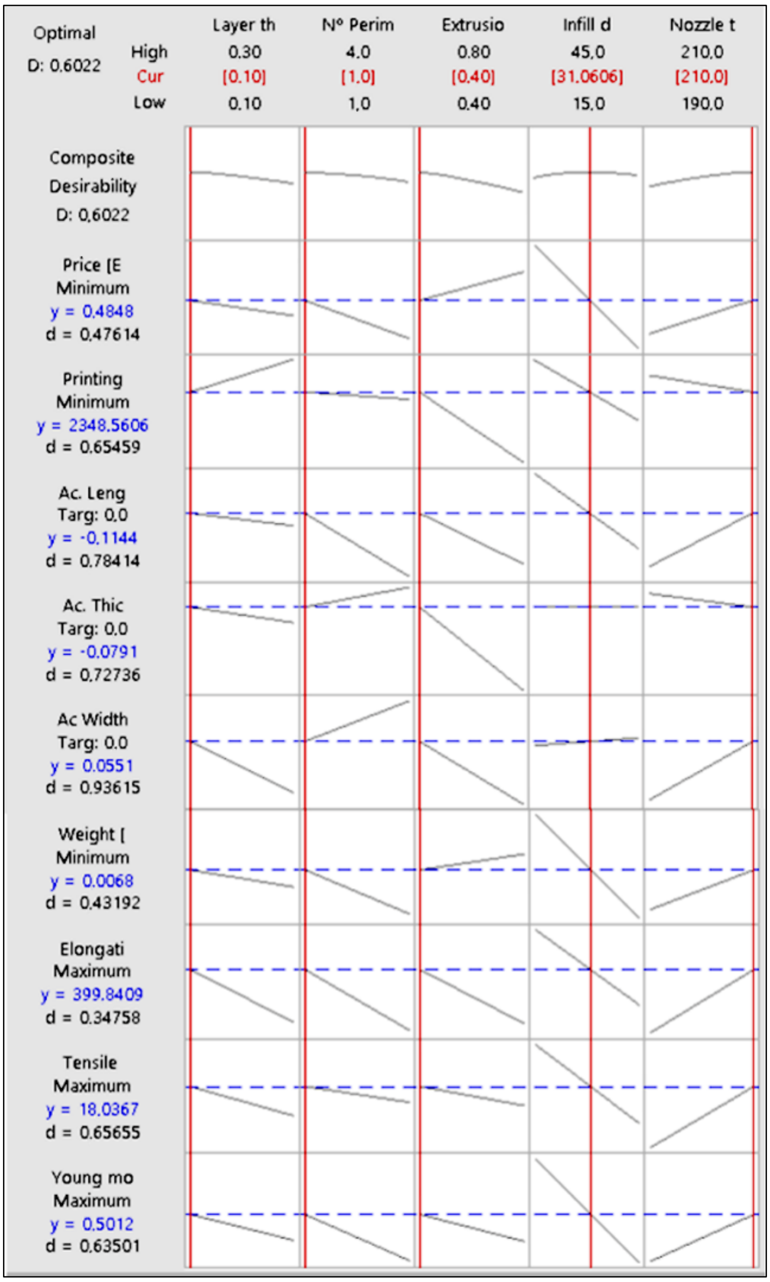

(c)

Figure S7. Composite desirability and individual desirabilities for all responses for (a) PLA, (b) PETG, (c) PHA.

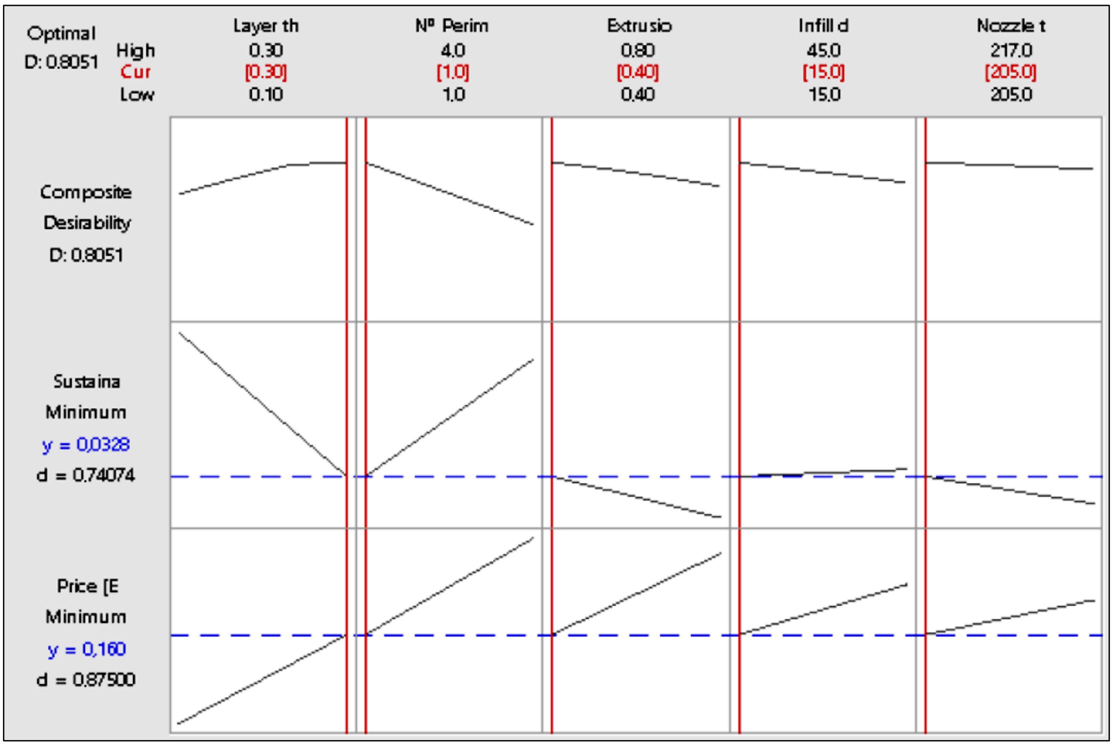

(a)

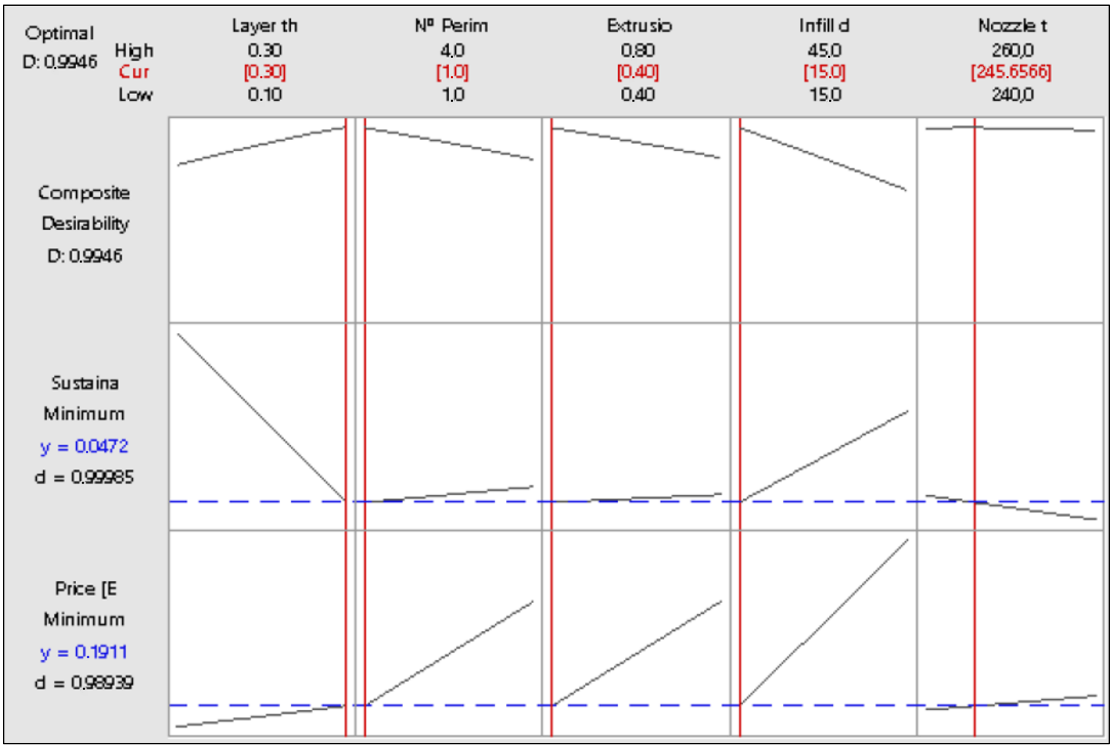

(b)

Figure S8. Optimization according to sustainability (CO2-eq) and filament price (EUR) for (a) PLA, (b) PETG material.
